# Carbohydrate concentration and type drive product selectivity to a mixture of volatile fatty acids or lactic acid in thermophilic mixed-culture fermentation

**DOI:** 10.1007/s00253-026-13806-0

**Published:** 2026-04-11

**Authors:** Laia Vulart, Néstor Izcara, Ángel Estévez, Enrique Peiro, Francesc Gòdia, Ramon Ganigué

**Affiliations:** 1 Departament d’Enginyeria Química, Biològica i Ambiental, Universitat Autònoma de Barcelona, 08193 Bellaterra (Cerdanyola del Vallès), Spain; 2 Center for Microbial Ecology and Technology (CMET), Ghent University, 9052 Ghent, Belgium; 3 Centre for Advanced Process Technology for Urban Resource Recovery (CAPTURE), 9052 Ghent, Belgium

**Keywords:** Carboxylate platform, Carboxylic acids, Glucose, Xylose

## Abstract

**Abstract:**

Carboxylic acids are key platform chemicals that can be biologically produced in mixed-culture fermentations. In these systems, product formation is determined by the microbial community composition, which is shaped by operational conditions. A general trend has been observed linking substrate availability to the production of either lactic acid or volatile fatty acids, but it remains unclear at which substrate concentration this shift occurs. This study investigates the effect of carbohydrate concentrations (from 0 to 670 mg COD·L^−1^) and type (hexoses and pentoses) on product selectivity and microbial community composition in thermophilic mixed-culture fermentations. Fermentation experiments were conducted in thermophilic reactors (55 °C, pH 5.3, HRT 4 days), fed either with glucose or xylose. Higher substrate concentrations (650–670 mg COD·L^−1^) favored lactic acid production, accounting for 60–76% of the soluble products. Lower carbohydrate concentrations reduced lactic acid production and increased volatile fatty acid concentrations. Volatile fatty acids became the main product under low substrate concentrations (continuous operation; 0 mg COD·L^−1^), with a selectivity of 83–97%. Despite this general trend, the substrate concentration at which the shift from lactic acid to volatile fatty acids occurred depended on the carbohydrate type. Microbial community analyses revealed *Thermoanaerobacterium* as the dominant genus in all reactors, with genera within the *Bacillaceae* family (putatively involved in lactic acid production) increasing in relative abundance under high carbohydrate concentrations. This suggests that the product shift resulted from both a metabolic shift within the dominant species and a change in the microbial community composition.

**Key points:**

- *Low carbohydrate concentrations favor VFA production.*
- *High carbohydrate concentrations promote lactic acid formation.*
- *Carbohydrate type influences the shift from lactic acid to VFAs.*

**Supplementary Information:**

The online version contains supplementary material available at 10.1007/s00253-026-13806-0.

## Introduction

Organic waste valorization plays a critical role in the transition toward a circular and resource-efficient bioeconomy. Until now, anaerobic digestion is the most common technology used to valorize organic waste in the form of biogas (Kleerebezem et al. 2015; Scarlat et al. 2018). However, biogas has limited economic value and its production through anaerobic digestion is highly reliant on subsidies (Scarlat et al. 2018). Several alternatives are being explored to generate higher-value products from organic waste (Baumann and Westermann 2016; Kleerebezem et al. 2015). For instance, the carboxylate platform targets the conversion of organic feedstocks (e.g., food waste, lignocellulosic biomass waste) into short-chain carboxylic acids using mixed microbial communities (Holtzapple et al. 2022). These compounds serve as platform chemicals that can be converted into a wide range of products, including biopolymers, biofuels, and green solvents (Ramos-Suarez et al. 2021). In this context, mixed-culture biotechnology offers a robust and cost-effective approach, as these processes do not require axenic conditions and can handle heterogeneous waste streams (Holtzapple et al. 2022).

In mixed cultures, the fermentation products are determined by the enriched microbial community, which is shaped by experimental conditions such as temperature, pH, and feeding regime (Holtzapple et al. 2022; Ramos-Suarez et al. 2021; Vázquez-Fernández et al. 2022). Among these, the feeding regime is a critical parameter, determining how, when, and in what amount the substrate is added to the system. For example, in continuous stirrer-tank reactors (CSTRs), substrate is supplied continuously and at the same rate that effluent is removed, enabling the system to reach steady-state conditions with stable substrate and product concentrations. If the system operates below its critical organic loading rate, substrate is completely consumed, resulting in low substrate concentrations in the reactor throughout operation. Conversely, sequencing batch reactors (SBRs) operate in cycles that include different phases: fill, react, decant, and idle. Substrate is added only during the fill phase, while no outflow occurs until the decant phase. As a result, substrate and product concentrations can fluctuate during the cycle (Ochoa 2019; Penia Kresnowati and Chen 2011). Therefore, the feeding regime directly influences how the substrate is supplied to the microorganisms. Despite experimental conditions varying widely between studies, a general trend has been observed between substrate supply and the resulting product profile (Rombouts et al. 2019; Vulart et al. 2025). In systems like CSTRs where substrate concentrations in the system remain low, the production of a mixture of volatile fatty acids (VFAs) was usually observed. By contrast, systems with high and/or intermittent substrate addition, such as SBRs, promoted the production of lactic acid (Contreras-Dávila et al. 2020; Liu et al. 2023; Vulart et al. 2025).

However, the complex nature of waste-derived substrates makes it difficult to assess how substrate concentrations drive this shift between lactic acid and VFA production. Real feedstocks often require an initial hydrolysis step to break down polymers into monomers accessible to microorganisms (Kleerebezem et al. 2015), which ultimately affects the concentration of bioavailable substrate, linking it to the hydrolysis kinetics. To isolate the effect of substrate concentration, studies using monomeric substrates can offer clearer insights by eliminating the variability introduced by hydrolysis. Glucose and xylose are the most abundant monomeric sugars in several types of organic residues (e.g., lignocellulosic biomass waste, food waste) (Narisetty et al. 2022) and are commonly considered as model carbohydrate monomers for hexoses and pentoses, respectively. To date, studies investigating the effect of glucose or xylose concentrations on product formation and microbial community dynamics remain limited. Rombouts et al. (2019) studied the effect of substrate supply by comparing continuous and sequencing batch operation using glucose and xylose as substrates under mesophilic conditions and pH 8. Their results show VFAs and ethanol as the main fermentation products under both regimes. However, the use of a defined mineral medium in their study may have constrained the growth of lactic acid bacteria, many of which are auxotrophic for several amino acids and vitamins and often require complex nutrients (Rombouts et al. 2020). Iglesias-Riobó et al. (2025) investigated the influence of the organic loading rate on xylose fermentation (37 °C, pH 6, complex medium) in two reactor configurations, a CSTR and a SBR. In both systems, VFAs were the main fermentation products. However, lactic acid was also produced when the reactors were overloaded and xylose was not completely consumed. In addition, the characterization of the SBR cycle revealed that lactic acid was produced and subsequently consumed in secondary fermentations (Iglesias-Riobó et al. 2025). Other studies investigating the effect of substrate concentrations have focused on varying the concentration of glucose or xylose in the influent under continuous operation, at both mesophilic (Hoelzle et al. 2021; Iglesias-Riobó et al. 2025; Temudo et al. 2008, 2009) and thermophilic conditions (Zhang et al. 2015). Those studies conducted with mineral media also reported the formation of VFAs and ethanol at different substrate concentrations (Temudo et al. 2008, 2009). In contrast, when a complex medium was used (Hoelzle et al. 2021; Zhang et al. 2015), a mixture of VFAs was the main fermentation product when substrate was fully consumed, whereas lactic acid accumulated under conditions of incomplete substrate consumption. Despite changes in influent substrate concentration, CSTRs operated below the critical organic loading rate of the system resulted in low residual glucose concentrations in the reactor, limiting insights into how substrate concentrations in the reactor determine microbial community composition and product profile.

Thermophilic systems may offer advantages over mesophilic conditions for understanding the primary metabolic pathways involved in fermentation processes. Lactic acid and ethanol are known electron donors in secondary fermentation processes, such as chain elongation reactions or lactate oxidation to butyrate, producing medium-chain carboxylic acids such as butyric and caproic acid (Agler et al. 2011; Spirito et al. 2014). These secondary fermentations may hinder the identification of the primary fermentation products, as well as the metabolic pathways through which these are formed. Thermophilic temperatures can limit the conversion of lactic acid and ethanol in secondary fermentations. To date, no thermophilic culture able to carry out ethanol chain elongation has been reported. Regarding lactic acid, Sakarika et al. (2023) observed butyric acid production from lactic acid at temperatures up to 50 °C, whereas an increase to 55 °C led to lactic acid accumulation, with no subsequent conversion to butyric acid. Contreras-Dávila et al. (2020) already detected lactic acid accumulation in a thermophilic reactor fermenting food waste at 50 °C, whereas the conversion of lactic acid to butyric acid and traces of caproic acid was observed when the temperature was reduced to 35 °C. Therefore, thermophilic temperatures (55 °C) were selected in this study to limit the further conversion of lactic acid and ethanol, thereby allowing a clearer distinction between direct sugar fermentation to VFAs and two-step pathways involving lactic acid or ethanol as intermediates.

The present study aims to (1) assess the impact of substrate concentrations (from 0 to 670 mg COD·L^−1^) on the production of lactic acid and VFAs during mixed-culture thermophilic fermentation of carbohydrates, (2) examine how different carbohydrates (glucose and xylose) affect the product spectrum, and (3) evaluate how these factors shape the microbial community. To address these objectives, three thermophilic (55 °C) stirred tank reactors were operated under sequencing batch or continuous modes using glucose or xylose as carbon sources. Improved understanding of microbial community dynamics and product formation in open fermentation systems can support the development of more efficient bioprocesses for the valorization of organic waste.

## Materials and methods

### Inoculum and substrate

Sludge obtained from a thermophilic anaerobic fermenter was used as the inoculum for reactors R1 and R3 (Table 1) (Vulart et al. 2025). This seed reactor was operated under continuous feeding at a temperature of 55 °C, pH of 5.3, hydraulic retention time (HRT) of 10 days, and sludge retention time (SRT) of 80–100 days. This fermenter was fed with a mix of 30% dry matter (DM) lettuce (*Lactuca sativa *var.* capitata*), 30% DM red beet (*Beta vulgaris *subsp.* vulgaris *var.* ruba*), 30% DM wheat straw (*Triticum aestivum*), and 10% DM toilet paper (Eco Planet, Carrefour, Belgium), and produced a mixture of VFAs as the main fermentation product (1.74 ± 0.17 g COD·L^−1^ acetic acid, 1.08 ± 0.21 g COD·L^−1^ butyric acid, 0.31 ± 0.07 g COD·L^−1^ caproic acid, and 0.46 ± 0.06 g COD·L^−1^ of other C3–C8 VFAs). Sludge was sieved through a 1-mm milling sieve to remove larger particles and incubated at 55 °C for 3 days before inoculation. Reactor R2 was inoculated with effluent collected from reactor R1.

**Table 1:** Reactor nomenclature, type of carbon source, concentration of carbon source in the influent (mg COD·L^−1^), reactor operation mode (including cycle length during sequencing batch operation), theoretical carbon source concentration at the beginning of the cycle in each reactor (mg COD·L^−1^), operation periods (days), and steady-state periods (days)

Reactor	Carbon source	Influent concentration (g COD·L^−1^)	Reactor operation	Initial reactor concentration per cycle (g COD·L^−1^)	Period (days)	Steady-state period (days)
R1	Glucose	18.2	SBR 3.0 h	610	0–47	28–42
			SBR 1.2 h	240	48–103	77–91
			Continuous	≈0	104–142	128–142
R2	Glucose	7.8	SBR 3.0 h	240	0–42	28–42
			SBR 1.2 h	100	43–76	54–76
			Continuous	≈0	77–106	90–106
R3	Xylose	21.4	SBR 3.0 h	670	0–61	40–51
			SBR 1.2 h	270	62–139	125–136
			Continuous	≈0	140–243	215–243

The composition of the medium is shown in Table S1. It was supplemented by a 7-vitamins solution, a trace elements SL10 solution, and a selenite and tungstate solution according to Table S2. Medium pH was then adjusted to 4.5 by adding 2M HCl. Carbon source (glucose or xylose) solutions were supplied according to the concentrations reported in Table 1. These concentrations were selected based on concentration and organic loading ranges commonly reported in previous studies employing glucose or xylose as carbon sources (Rombouts et al. 2020; Temudo et al. 2007, 2008; Zhang et al. 2015). Higher influent concentrations were avoided to prevent potential toxicity associated with high carboxylic acid concentrations (López-Garzón and Straathof 2014; Ramos-Suarez et al. 2021; Wilbanks and Trinh 2017; Xiao et al. 2016) .

### Experimental set-up

Fermentation experiments were conducted in three jacketed reactors with a working volume of 1 L. All reactors were run under anaerobic and thermophilic conditions with an operational temperature of 55 °C. Anaerobic conditions were ensured by flushing N_2_ at the start of the experiment and temperature was maintained using a water bath circulator and a built-in water jacket. The pH was set to 5.3 and maintained using an automatic pH control system that dosed 2 M NaOH as needed. Both HRT and SRT were maintained at 4 days. All reactors were mixed with magnetic stirrers at 300 rpm to ensure homogeneity.

Reactors R1 and R3 were inoculated with 100 mL of sludge and initially operated in batch mode for 24 h, with the addition of 1 g·L^−1^ of the correspondent carbon source (Table 1). After, the reactors were switched to sequencing batch mode. Reactor R2 was inoculated with the effluent of R1 as inoculum and operated directly in sequencing batch mode. All reactors were operated with a cycle length of 3.0 h during the first period of the experiment, and 1.2 h for the second period (Table 1). Decant and fill phases were carried out during the first minutes of each cycle. By modifying the cycle length while maintaining a constant HRT, it was possible to adjust the concentration of the carbon source present in the reactor at the beginning of each cycle. It should be noted that the total organic load per HRT was kept constant throughout the experiment; only its distribution within each HRT was modified. Longer cycles resulted in less frequent but higher substrate concentration peaks, whereas shorter cycles led to more frequent and lower concentration peaks (Fig. S1). The theoretical carbon source concentrations for each reactor and cycle are detailed in Table 1 and were confirmed by high-performance liquid chromatography (HPLC) (Fig. S1). In the third period of the experiment, the reactors were operated under continuous mode (Table 1). Since there was complete substrate utilization, the carbon source concentration in the reactors was close to 0 mg COD·L^−1^. Steady-state was defined as a variation of less than 15% in the concentration of the main fermentation products (lactic, acetic, and/or butyric acid) for at least 3 HRTs (Table 1).

### Analytical methods

#### Chemical analyses

Influent samples (medium) were taken once a week to measure the soluble chemical oxygen demand (COD) derived from adding yeast extract and tryptone. Effluent and gas samples were taken two/three times per week to determine total suspended solids (TSS) and volatile suspended solids (VSS), soluble COD, and main fermentation products. Effluent samples were centrifuged for 20 min at 7197 rcf; the supernatant was then filtered through a 0.20-μm Chromafil Xtra syringe filters (MACHEREY-NAGEL, Düren, Germany) and stored at −20 °C until analysis. Pellet was resuspended appropriately to analyze TSS and VSS.

TSS and VSS were quantified based on the weight difference of a sample before and after drying at 105 °C and 550 °C, respectively, in accordance with Standard Method 2540 (“2540 SOLIDS,” 2017). Soluble COD was measured using Nanocolor test kits (MACHEREY-NAGEL, Düren, Germany). Lactic and acetic acid were analyzed by ion chromatography (IC) using a 930 Compact IC Flex system (Metrohm, Herisau, Switzerland) with Metrohm CO_2_ Suppression and an 850 IC conductivity detector, and equipped with a Metrosep Organic Acids 250/7.8 column and a Metrosep Organic Acids Guard/4.6 guard column. C3 to C6 carboxylic acids were determined using gas chromatography (GC) on a Shimadzu GC-2014 system (Kyoto, Japan) equipped with a DB-FFAP 123–3232 column (30 m × 0.32 mm × 0.25 μm) (Agilent, Santa Clara, USA) and a flame ionization detector (Shimadzu, Kyoto, Japan). The carrier gas was N_2_ at a flow rate of 2.49 mL·min^−1^. Prior to analysis, samples were treated with sulfuric acid and sodium chloride, followed by extraction with diethyl ether. 2-Methyl hexanoic acid was added as an internal standard during extraction. Ethanol, glucose, and xylose were quantified by HPLC using a Shimadzu LC-2030 i-Series system (Kyoto, Japan), equipped with an Aminex HPC-87H column (Bio-rad, Hercules, USA), a Micro-Guard Cation H Cartridge guard column (Bio-rad, Hercules, USA), a SPD-40/40V UV/Vis detector (Shimadzu, Kyoto, Japan), and a RID-20A refractive index detector (Shimadzu, Kyoto, Japan). Gas composition in the reactor headspace was analyzed by GC using a Compact GC 4.0 (Global Analyser Solutions, Breda, The Netherlands), equipped with a Molsieve 5 A pre-column and Porabond Q column (CH_4_, O_2_, H_2_, and N_2_), and a Rt-Q-Bond pre-column and column (CO_2_). Gas concentrations were determined with a thermal conductivity detector. The carrier gases used were N_2_ and He.

#### Molecular analyses

16S rRNA gene sequencing was conducted on effluent samples of each reactor previously stored at −20 °C. DNA extraction was performed with the Dneasy PowerSoil Pro Kit (Qiagen, Hilden, Germany). The bead-beating step was carried out using a PowerLyzer 24 Homogenizer (Qiagen, Hilden, Germany) at 2000 rpm for 5 min. DNA was eluted with 100 µL C6 (Tris buffer).

Genomic DNA extract was sent out to LGC Genomics (Berlin, Germany) for library preparation and sequencing on an Illumina Miseq platform by targeting the V3–V4 hypervariable region of the 16S rRNA gene. The polymerase chain reaction (PCR) mix included 1 µL of DNA extract, 15 pmol of both the forward primer and reverse primer in 20 µL volume of MyTaq buffer containing 1.5 units MyTaq DNA polymerase (Meridian Bioscience, Cincinnati, USA), and 2 µL of BioStab II PCR Enhancer (Sigma Aldrich, Saint Louis, USA). Primers 341F 5′-CCTACGGGNGGCWGCAG-3′ and 785Rmod 5′-GACTACHVGGGTATCTAAKCC-3′ were used. The reverse primer was adapted from Klindworth et al. (2013) to increase coverage. PCR was run for 30 cycles using the following parameters: predenaturation at 96 °C for 2 min; 96 °C for 15 s, 50 °C for 30 s, 70 °C for 90 s. DNA concentration of amplicons of interest was determined by gel electrophoresis. Next, about 20 ng amplicon DNA of each sample were pooled for up to 48 samples carrying different barcodes. Amplicon pools were purified with one volume AMPure XP beads (Agencourt Bioscience, Beverly, USA) to remove primer dimer and other small mispriming products, followed by an additional purification on MinElute columns (Qiagen, Hilden, Germany). About 100 ng of each purified amplicon pool DNA was used to construct Illumina libraries via the adaptor ligation using the Ovation Rapid DR Multiplex System 1–96 (NuGEN Technologies, San Francisco, USA). Illumina libraries were pooled and size-elected by preparative gel electrophoresis, and sequencing was performed on an Illumina MiSeq using V3 Chemistry (Illumina Inc, San Diego, USA).

#### Data analysis

Sequencing data was analyzed using R version 4.4.2 (2024-10-31). The DADA2 R package was used to process the amplicon sequence data according to the pipeline tutorial (Callahan et al. 2016). As an initial quality control step, primer sequences were removed and reads were truncated based on a quality score threshold (truncQ = 2). Additional filtering steps were conducted to exclude reads containing any ambiguous base calls or high expected errors (maxEE = 2, 2). A concise overview of read quality metrics regarding both the raw and filtered sequences is provided in Fig. S2. After dereplication, unique reads were further denoised using the Divisive Amplicon Denoising Algorithm error estimation algorithm and the selfConsist sample inference algorithm (with the option pooling = TRUE). The resulting error rates were inspected and, upon validation, the denoised reads were merged. Chimeric sequences were identified and removed. The number of reads retained at each processing step is summarized in Table S3. The resulting amplicon sequence variant (ASV) table was used for taxonomy assignment employing the Naive Bayesian Classifier and the DADA2 formatted Silva v138.1 (Quast et al. 2013). Any ASV with a total abundance across all samples less than or equal to 1 were excluded. Two mock community controls were also included in the sequencing run to verify run quality and pipeline performance (data not shown).

The raw fastq files of the 16S amplicon sequences have been deposited in the National Center for Biotechnology Information (NCBI) database, under the accession number PRJNA1347372.

Alpha- and beta-diversity analyses were performed in R version 4.5.2 (2025-10-31) using the phyloseq and vegan packages. Alpha-diversity was quantified using the Shannon index based on the ASV abundance table. Beta-diversity was assessed using Bray-Curtis dissimilarity, and differences in community composition were visualized using principal coordinates analysis (PCoA).

### Calculations

The COD balance of the different soluble fermentation products was calculated by dividing the soluble COD and biomass (VSS) of the effluent by the soluble COD of the influent (1). For VSS conversion to COD, it was assumed a COD equivalent of 1.37 g COD·g^−1^ (Roels 1980). The COD of the off-gas effluent was not considered in this equation, as gas production could not be measured during the experiment.

1 $$COD\;balance \left(\mathrm{\%}\right)=\frac{{COD}_{sol, eff} (\text{mg COD}\cdot {\mathrm{L}}^{-1})+{VSS}_{eff} (\text{mg COD}\cdot {\mathrm{L}}^{-1})}{{COD}_{sol, inf} (\text{mg COD}\cdot {\mathrm{L}}^{-1})}\times 100$$

The selectivity of the different soluble fermentation products was calculated by dividing the concentration of each individual product in COD by the sum of all products (2).

2 $$Fermentation\;product\;selectivity \left({\% COD}\right)=\frac{{C}_{FP, eff} (\text{mg COD}\cdot {\mathrm{L}}^{-1})}{{C}_{Total FP, eff} (\text{mg COD}\cdot {\mathrm{L}}^{-1})}\times 100$$

The yield of the different fermentation products was calculated by dividing the amount of each product in COD by the influent COD consumed in the system (3). Because gas production could not be measured during the experiment, the amount of H_2_ produced was estimated as a theoretical value based on stoichiometric relationships that reflect the distribution of reducing equivalents at the pyruvate-acetyl-CoA node. Specifically, it was assumed 1 mol H_2_ per mol of acetic acid, 2 mol H_2_ per mol of butyric acid, and 1 mol H_2_ per mol of ethanol (Temudo et al. 2007). Therefore, H_2_ yield is reported as a theoretical value.

3 $$Product\;yield \left(\text{g COD}\cdot {\mathrm{g}}^{-1}\text{ COD}\right)=\frac{{M}_{FP, eff} (\text{g COD})}{Consumed\;COD (\text{g COD})}$$

The consumed COD was calculated according to Eq. 4. The influent contained both a carbon source (glucose or xylose) and yeast extract and tryptone (referred to as “medium”). To account for this, Eq. 4 estimates the COD consumed from both the carbon source and yeast extract and tryptone.

4 $$Consumed\;COD \left(\text{g COD}\right)={\Delta M}_{C source} \left(\text{g COD}\right)+\Delta COD\;medium \left(\text{g COD}\right)$$

The amount of carbon source consumed was determined as the difference between the carbon source in the influent and its residual in the effluent (5).

5 $${\Delta M}_{C source} \left(\text{g COD}\right)={M}_{C source, inf} \left(\text{g COD}\right)-{M}_{C source, eff} \left(\text{g COD}\right)$$

Similarly, the consumed COD from the medium was calculated as the difference between the soluble COD provided by yeast extract and tryptone in the influent (measured experimentally) and the unidentified COD in the effluent (6). The latter was determined by subtracting the COD of identified products (lactic acid, C2-C6 VFAs, and ethanol) from the soluble COD of the effluent, according to Eq. 7.

6 $$\Delta COD medium \left(\text{g COD}\right)={COD}_{sol, YE+T inf} \left(\text{g COD}\right)-{COD}_{sol, unidentified eff} (\text{g COD})$$

7 $${COD}_{sol, unidentified\;eff} \left(\text{g COD}\right)={COD}_{sol, eff} (\text{g COD})-{M}_{FP, eff} (\text{g COD})$$

The soluble COD from the medium (6) was included in Eq. 4, and consequently in the yield calculation (Eq. 3), only when the result from Eq. 6 was a positive value. This indicated that part of the yeast extract and tryptone contributed to the formation of fermentation products.

## Results

### Metabolite distribution in response to glucose concentration

To evaluate how glucose concentration in the reactor influenced the product profile, reactors R1 and R2 were operated with glucose as the main carbon source. The concentration of glucose in the influent was 18.2 g COD·L^−1^ for reactor R1 and 7.8 g COD·L^−1^ for reactor R2. The glucose concentrations tested in the reactor were 610 mg COD·L^−1^, 240 mg COD·L^−1^, and 0 mg COD·L^−1^ in reactor R1, and 240 mg COD·L^−1^, 100 mg COD·L^−1^, and 0 mg COD·L^−1^ in reactor R2. These values represent theoretical glucose concentrations in the reactor at the start of each SBR cycle or under continuous operation due to complete substrate consumption and were experimentally confirmed by HPLC (Fig. S1).

**Fig. 1:**
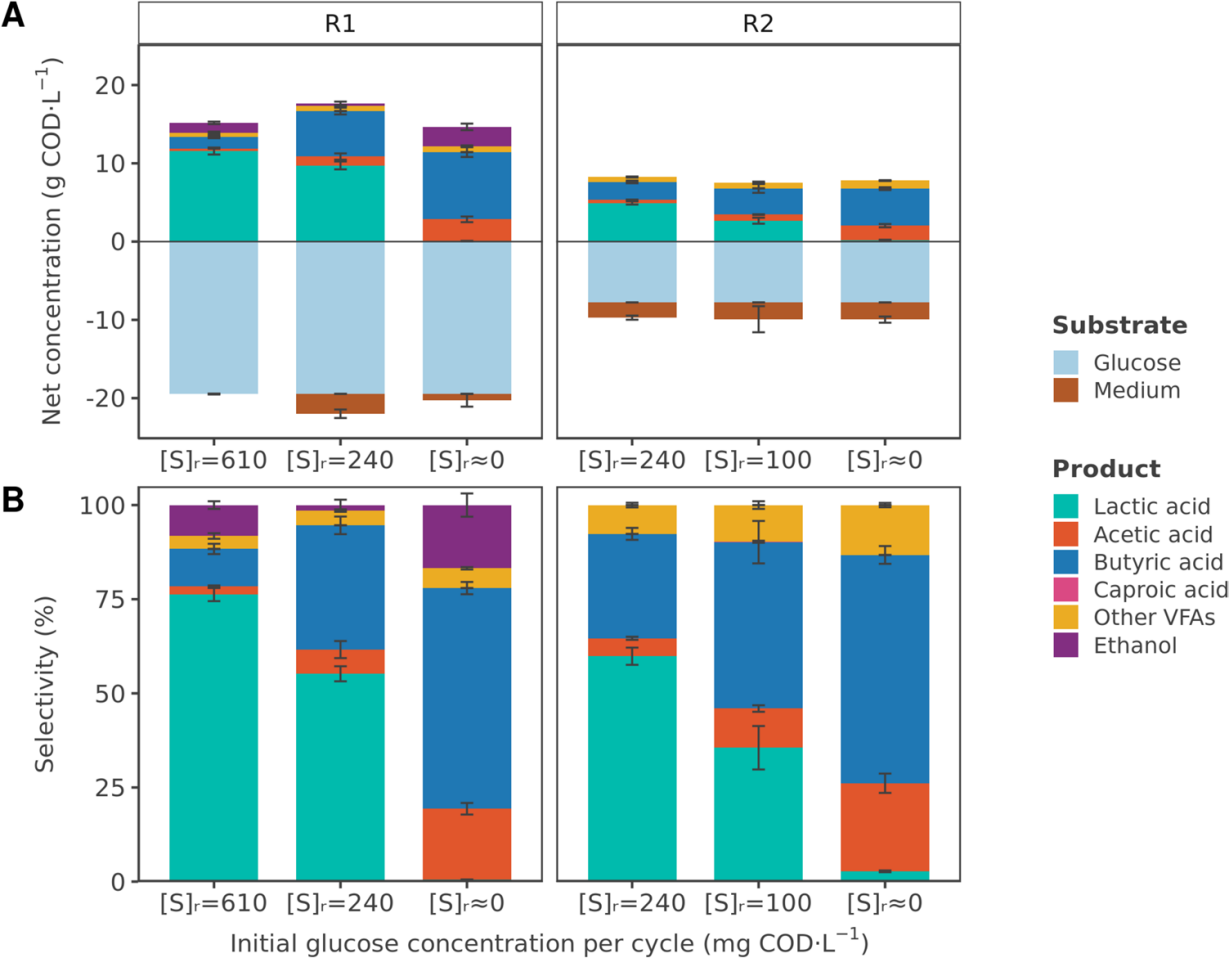
**A** Average net concentrations (mg COD·L^−1^) and **B** selectivity (%) of the soluble substrates and fermentation products during steady-state periods in reactor R1, fed with 18.2 g COD·L^−1^ of glucose, and reactor R2, fed with 7.8 g COD·L^−1^ of glucose. The tested reactor glucose concentrations ([S]_r_) were 610 mg COD·L^−1^, 240 mg COD·L^−1^, 100 mg COD·L^−1^, and 0 mg COD·L^−1^. “Medium” refers to the COD contribution from consumed yeast extract and tryptone. “Other VFAs” include propionic acid, isobutyric acid, valeric acid, isovaleric acid, and isocaproic acid. Gas and biomass are excluded from the products

High glucose concentrations favored lactic acid production over a mixture of VFAs (Fig. 1, Fig. S3). With an initial glucose concentration of 610 mg COD·L^−1^ at the beginning of the SBR cycle, lactic acid represented 76 ± 2% of the total soluble fermentation products in COD (11.7 ± 0.7 g COD·L^−1^), while VFAs only accounted for 16 ± 1% (2.4 ± 0.2 g COD·L^−1^). Among the VFAs, butyric acid was the most abundant, with 1.5 ± 0.2 g COD·L^−1^. Ethanol was also present in the reactor at concentrations of 1.3 ± 0.2 g COD·L^−1^ (selectivity of 8 ± 1%). The reactor headspace contained 28 ± 4% of H_2_ and 56 ± 6% of CO_2_ (Table 2).

**Table 2:** Chemical oxigen demand (COD) balance, product yield, and gas composition in the reactor headspace measured in reactor R1 and reactor R2 at different reactor glucose concentrations. Values represent the average and standard deviation of measurements taken during each steady-state period. COD balances refer only to quantified soluble products and biomass and do not include H_2_ production. The H_2_ yield corresponds to a theoretical value, calculated stoichiometrically from the quantified soluble fermentation products (acetic acid, butyric acid, and ethanol) (Temudo et al. 2007). “Other VFAs” include propionic acid, isobutyric acid, valeric acid, isovaleric acid, and isocaproic acid. “VSS” refers to volatile suspended solids and represents biomass

Reactor		R1			R2		
Carbon source		Glucose			Glucose		
Influent concentration (g COD·L^−1^)		18.2			7.8		
Reactor operation		SBR 3.0 h	SBR 1.2 h	Continuous	SBR 3.0 h	SBR 1.2 h	Continuous
Initial substrate concentration per cycle (mg COD·L^−1^)		610	240	≈0	240	100	≈0
**COD balance (%)**		85 ± 4	79 ± 7	81 ± 5	89 ± 4	87 ± 8	85 ± 3
**Product yield (g COD · g**^**-1**^ **COD)**	Lactic acid	0.56 ± 0.04	0.39 ± 0.03	0.00 ± 0.00	0.47 ± 0.03	0.27 ± 0.05	0.02 ± 0.00
	Total VFAs	0.11 ± 0.01	0.31 ± 0.04	0.58 ± 0.04	0.32 ± 0.03	0.48 ± 0.08	0.71 ± 0.03
	Acetic acid	0.02 ± 0.00	0.05 ± 0.02	0.13 ± 0.01	0.04 ± 0.00	0.08 ± 0.01	0.17 ± 0.02
	Butyric acid	0.07 ± 0.01	0.24 ± 0.03	0.41 ± 0.02	0.22 ± 0.02	0.33 ± 0.07	0.45 ± 0.02
	Caproic acid	0.00 ± 0.00	0.00 ± 0.00	0.00 ± 0.00	0.00 ± 0.00	0.00 ± 0.00	0.00 ± 0.00
	Other VFAs	0.03 ± 0.00	0.03 ± 0.00	0.04 ± 0.00	0.06 ± 0.01	0.07 ± 0.01	0.10 ± 0.01
	Ethanol	0.06 ± 0.01	0.01 ± 0.01	0.12 ± 0.02	0.00 ± 0.00	0.00 ± 0.00	0.00 ± 0.00
	VSS	0.06 ± 0.01	0.05 ± 0.01	0.06 ± 0.01	0.06 ± 0.01	0.07 ± 0.02	0.07 ± 0.02
	H_2_ (theoretical)	0.03 ± 0.00	0.06 ± 0.01	0.14 ± 0.01	0.05 ± 0.01	0.09 ± 0.01	0.13 ± 0.01
**Gas composition (%)**	H_2_	28 ± 4	50 ± 4	47 ± 2	43 ± 1	50 ± 3	57 ± 6
	CO_2_	56 ± 6	46 ± 2	46 ± 3	51 ± 1	48 ± 3	43 ± 1

A decrease in substrate concentrations resulted in lower lactic acid production and a concurrent increase in VFA production (Fig. 1, Fig. S3, Fig. S4). The initial glucose concentration of 240 mg COD·L^−1^ was tested in both reactor R1 and reactor R2, which differed in the concentration of glucose in the influent (18.2 g COD·L^−1^ for reactor R1 and 7.8 g COD·L^−1^ for reactor R2). In reactor R1, lactic acid selectivity decreased to 55 ± 2% (9.7 ± 0.5 g COD·L^−1^), while VFAs increased to 43 ± 2% (7.6 ± 0.5 g COD·L^−1^). A similar product distribution was observed in reactor R2, in which lactic acid represented 60 ± 2% (4.9 ± 0.2 g COD·L^−1^) of the total soluble products, while VFAs accounted for 40 ± 2% (3.3 ± 0.2 g COD·L^−1^). Product yields were also comparable between the two systems (Table 2). Lactic acid and VFAs were produced at 0.39 ± 0.03 and 0.31 ± 0.04 g COD·g^−1^ COD of influent in reactor R1, and 0.47 ± 0.03 and 0.32 ± 0.03 g COD·g^−1^ COD of influent in reactor R2. The headspace of reactor R1 was composed of 50 ± 4% of H_2_ and 46 ± 2% of CO_2_ (Table 2), while that of reactor R2 contained H_2_ and CO_2_ at 43 ± 1% and 51 ± 1%, respectively. In both reactors, consumption of part of the yeast extract and/or tryptone present in the influent was observed (0.8–2.5 g COD·L^−1^), potentially contributing to the formation of fermentation products (Fig. 1). Additionally, an initial glucose concentration of 100 mg COD·L^−1^ was tested in reactor R2, resulting in a further decrease in lactic acid selectivity to 36 ± 6% (2.7 ± 0.4 g COD·L^−1^), while VFAs represented 64 ± 6% (4.9 ± 0.7 g COD·L^−1^) of the soluble fermentation products (Fig. 1, Fig. S4). The proportion of H_2_ in the reactor headspace increased to 50 ± 3%, while CO_2_ remained at 48 ± 3% (Table 2).

During the last experimental period, both reactors were operated under continuous mode, which translated into the concentration of glucose in the reactor being close to 0 mg COD·L^−1^. A mixture of VFAs was the main product in both systems (Fig. 1, Fig. S3, Fig. S4). In reactor R1, the selectivity of total VFAs was 83 ± 3%, with a concentration of 12.1 ± 1.1 g COD·L^−1^. Acetic acid (2.8 ± 0.4 g COD·L^−1^) and butyric acid (8.6 ± 0.6 g COD·L^−1^) were the main fermentation products. Ethanol was also detected at concentrations of 2.5 ± 0.4 g COD·L^−1^ (17 ± 3% selectivity). The reactor headspace contained 47 ± 2% of H_2_ and 46 ± 3% of CO_2_ (Table 2). In reactor R2, VFA selectivity reached 97 ± 0%, with a total VFA concentration of 7.6 ± 0.1 g COD·L^−1^. Acetic and butyric acid were also the predominant VFAs, with concentrations of 1.8 ± 0.2 g COD·L^−1^ and 4.7 ± 0.2 g COD·L^−1^, respectively. In contrast to reactor R1, ethanol was not produced under continuous feeding. The reactor headspace contained H_2_ and CO_2_ at 57 ± 6% and 43 ± 1%, respectively (Table 2).

### Impact of carbohydrate type on lactic acid and VFA selectivity

Reactor R3 was operated with xylose (21.4 g COD·L^−1^) as the main carbon source to study whether the resulting product profile depended on the type of carbohydrate. Xylose was chosen as a model carbohydrate for pentoses due to its abundance in lignocellulosic biomass and its relevance in bioconversion processes. The xylose concentrations tested in the reactor were 670 mg COD·L^−1^ and 270 mg COD·L^−1^ during the first and second experimental periods. During the third period, xylose concentrations in the reactor were close to 0 mg COD·L^−1^ due to complete substrate consumption under continuous operation. These theoretical values were confirmed by HPLC analysis (Fig. S1).

**Fig. 2:**
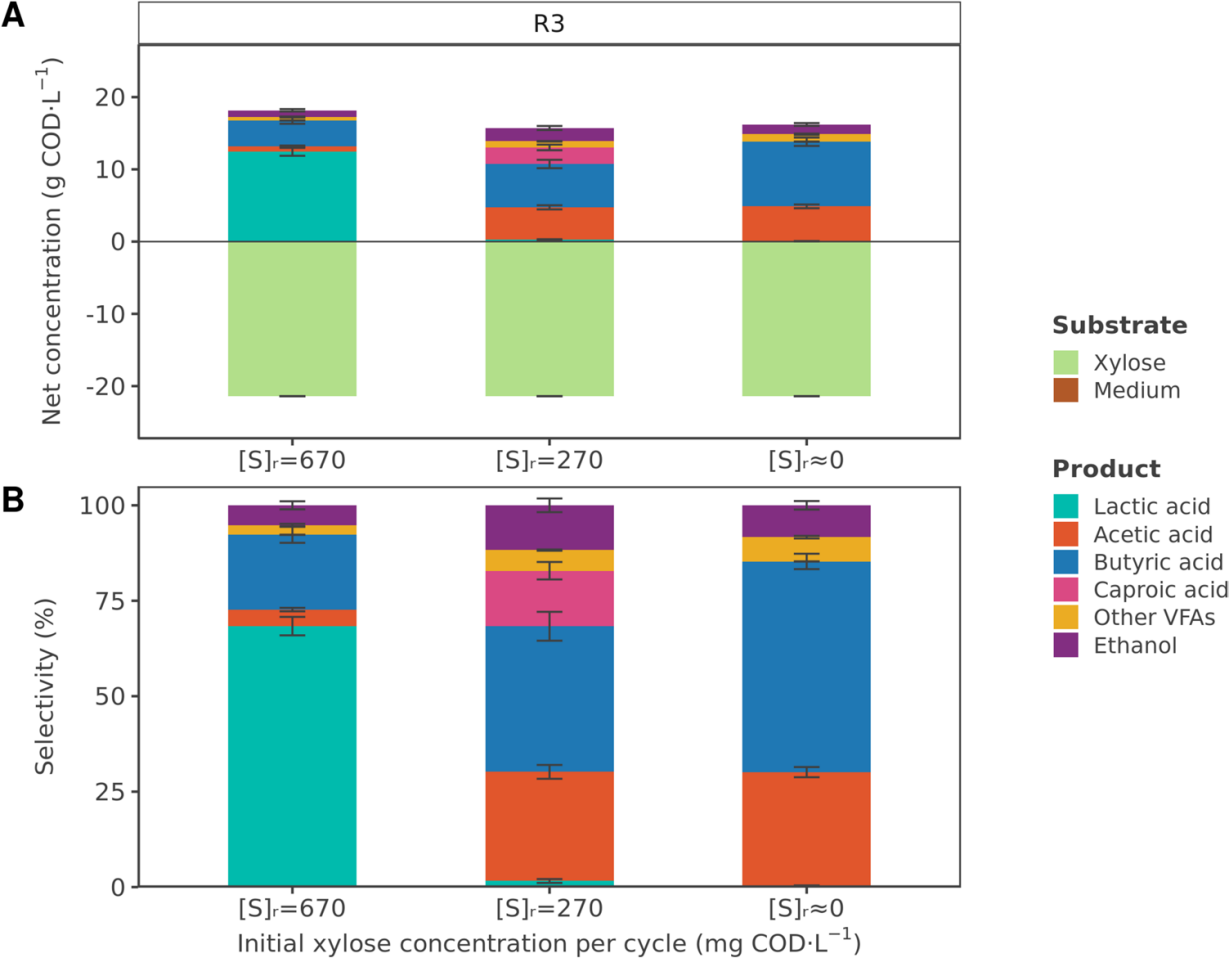
**A** Average net concentrations (mg COD·L^−1^) and **B** selectivity (%) of the soluble substrates and fermentation products during steady-state periods in reactor R3, fed with 21.4 g COD·L^−1^ of xylose. The tested reactor xylose concentrations ([S]_r_) were 670 mg COD·L^−1^, 270 mg COD·L^−1^, and 0 mg COD·L^−1^. “Medium” refers to the COD contribution from consumed yeast extract and tryptone. “Other VFAs” include propionic acid, isobutyric acid, valeric acid, isovaleric acid, and isocaproic acid. Gas and biomass are excluded from the products

The results indicate that higher substrate concentrations also favored lactic acid production over a mixture of VFAs when using xylose as the main carbon source (Fig. 2, Fig. S5). With a xylose concentration of 670 mg COD·L^−1^ in the reactor, lactic acid accounted for 68 ± 2% (12.4 ± 0.5 g COD·L^−1^) of the total soluble fermentation products, whereas VFAs represented 26 ± 3% (4.8 ± 0.6 g COD·L^−1^). Similar to experiments with glucose, butyric acid was the predominant VFA, reaching 3.6 ± 0.5 g COD·L^−1^. Ethanol was also produced at 0.9 ± 0.2 g COD·L^−1^ (5 ± 1% selectivity). The reactor headspace contained 41 ± 6% of H_2_ and 49 ± 2% of CO_2_ (Table 3).

**Table 3:** Chemical oxigen demand (COD) balance, product yield, and gas composition in the reactor headspace measured in reactor R3 at different reactor xylose concentrations. COD balances refer only to quantified soluble products and biomass and do not include H_2_ production. The H_2_ yield corresponds to a theoretical value, calculated stoichiometrically from the quantified soluble fermentation products (acetic acid, butyric acid, and ethanol) (Temudo et al. 2007). “Other VFAs” include propionic acid, isobutyric acid, valeric acid, isovaleric acid, and isocaproic acid. “VSS” refers to volatile suspended solids and represents biomass

Reactor		R3		
Carbon source		Xylose		
Influent concentration (g COD·L^−1^)		21.4		
Reactor operation		SBR 3.0 h	SBR 1.2 h	Continuous
Initial substrate concentration per cycle (mg COD·L^−1^)		670	270	≈0
**COD balance (%)**		90 ± 6	81 ± 3	78 ± 3
**Product yield (g COD · g**^**-1**^ **COD)**	Lactic acid	0.55 ± 0.06	0.01 ± 0.00	0.00 ± 0.00
	Total VFAs	0.21 ± 0.03	0.59 ± 0.02	0.63 ± 0.02
	Acetic acid	0.03 ± 0.00	0.20 ± 0.02	0.21 ± 0.01
	Butyric acid	0.16 ± 0.02	0.26 ± 0.03	0.38 ± 0.02
	Caproic acid	0.00 ± 0.00	0.10 ± 0.01	0.00 ± 0.00
	Other VFAs	0.02 ± 0.00	0.04 ± 0.00	0.04 ± 0.00
	Ethanol	0.04 ± 0.01	0.08 ± 0.01	0.06 ± 0.01
	VSS	0.07 ± 0.02	0.08 ± 0.01	0.05 ± 0.00
	H_2_ (theoretical)	0.05 ± 0.00	0.12 ± 0.01	0.14 ± 0.01
**Gas composition (%)**	H_2_	41 ± 6	47 ± 4	56 ± 3
	CO_2_	49 ± 2	43 ± 4	45 ± 2

When the initial xylose concentration per cycle was reduced to 270 mg COD·L^−1^, lactic acid concentrations decreased to nearly undetectable levels (0.3 ± 0.1 g COD·L^−1^) and a mixture of VFAs became the predominant fermentation product (Fig. 2, Fig. S5), accounting for 87 ± 2% (13.6 ± 0.5 g COD·L^−1^) of the soluble fermentation products. Acetic and butyric acid were detected at 4.5 ± 0.3 g COD·L^−1^ and 6.0 ± 0.6 g COD·L^−1^, respectively, while caproic acid reached 2.3 ± 0.4 g COD·L^−1^. Ethanol was also produced at a concentration of 1.8 ± 0.3 g COD·L^−1^, representing 12 ± 2% of the soluble fermentation products. The percentage of H_2_ in the reactor headspace increased to 47 ± 4%, while the percentage of CO_2_ remained at 45 ± 2% (Table 3).

Under continuous operation, xylose concentration in the reactor was around 0 mg COD·L^−1^ due to complete substrate consumption. During this period, VFAs remained the primary products, representing 91 ± 1% of the soluble fermentation products (Fig. 2, Fig. S5). The product profile was similar to the previous period, except that caproic acid was no longer detected, and butyric acid increased to 8.9 ± 0.6 g COD·L^−1^. In the reactor headspace, H_2_ increased further to 56 ± 3%, while CO_2_ was measured at 45 ± 2% (Table 3).

### Microbial community characterization

Representative microbial samples from each reactor and condition at steady-state were analyzed using 16S rRNA gene amplicon sequencing. Inoculum samples were also analyzed for comparison (Fig. S6).

**Fig. 3:**
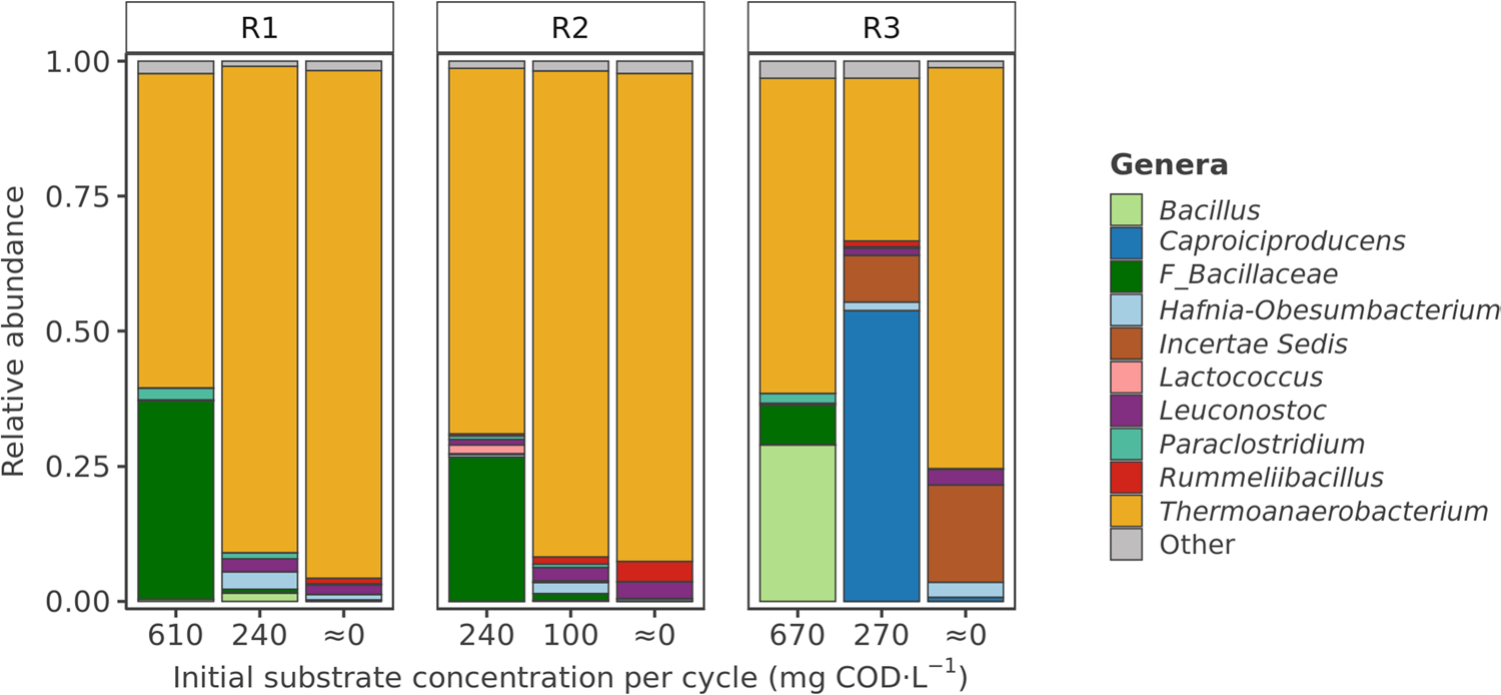
Relative abundance of bacteria at the genus level in reactors R1, R2, and R3, fed with 18.2 g COD·L^−1^ of glucose, 7.8 g COD·L^−1^ of glucose, and 21.4 g COD·L^−1^ of xylose, respectively, at different reactor substrate concentrations. For reactor R1, samples 610, 240, and ≈0 correspond to days 42, 91, and 142, respectively. For reactor R2, samples 240, 100, and ≈0 correspond to days 42, 76, and 106. For reactor R3, samples 670, 270, and ≈0 correspond to days 51, 139, and 243, respectively

The results demonstrated that both substrate concentration and carbohydrate type significantly shaped the bacterial microbial community (Fig. 3, Fig. S7). *Thermoanaerobacterium* was the dominant genus across all reactors, with a relative abundance above 50% in most experimental phases. An increase in the relative abundance of this genus was observed under lower reactor substrate concentrations, regardless of whether the substrate was glucose or xylose. In the glucose-fed reactors, the relative abundance of *Thermoanaerobacterium* was 67.6% in reactor R1 with an initial substrate concentration of 610 mg COD·L^−1^. This genus increased to 90.3% in reactor R1 and to 94.0% in reactor R2 under continuous operation, when the substrate concentration in the reactors was close to 0 mg COD·L^−1^. In reactor R3, which was fed with xylose, the relative abundance of *Thermoanaerobacterium* was 58.2% with an initial substrate concentration of 670 mg COD·L^−1^, and it increased to 74.2% under continuous feeding (0 mg COD·L^−1^).

Although this genus dominated the bacterial microbial community under most conditions, different ASVs of *Thermoanaerobacterium* were present at different relative abundances depending on the carbohydrate concentration and type (Fig. S7). Basic Local Alignment Search Tool (BLAST) searches for each ASV were performed against the NCBI nucleotide database (Table S4). ASVs 1, 5, and 7 had a 100.0% BLAST similarity to *Thermoanaerobacterium thermosaccharolyticum*. ASV2 had a 100% similarity to both *T. thermosaccharolyticum* and *Thermohydrogenium kirishiense*, while ASV4 matched the same species with 99.3% similarity. Additionally, ASVs 8 and 12 had a 99.8% and 100.0% similarity to both *Thermoanaerobacterium xylanolyticum* and *Thermoanaerobacterium calidifontis*, respectively. ASV22 was identified as both *T. thermosaccharolyticum* and *Thermoanaerobacterium aotearoense* with a 100.0% BLAST similarity.

Besides *Thermoanaerobacterium*, *Bacillus* and other genera within the *Bacillaceae* family were also present in all reactors under higher substrate concentrations (Fig. 3). In contrast to *Thermoanaerobacterium*, the relative abundance of genera within the *Bacillaceae* family increased as the initial substrate concentration in the reactor became higher, regardless of the type of carbohydrate fed. With an initial glucose concentration of 610 mg COD·L^−1^ in reactor R1, the relative abundance of genera within this family reached 26.7%, while a relative abundance of 36.3% was observed with an initial xylose concentration of 670 mg COD·L^−1^ in reactor R3. *Bacillus* ASV11 was identified as *Heyndrickxia coagulans* with a BLAST similarity of 100.0%, a thermophilic homofermentative lactic acid bacterium (Table S4) (Payot et al. 1999).

In contrast to the reactors fed with glucose, *Caproiciproducens* was the dominant genus in the xylose-fed reactor with an initial substrate concentration of 270 mg COD·L^−1^, with a relative abundance of 53.8% (Fig. 3). The genus *Caproiciproducens* includes strictly anaerobic bacteria that can convert carbohydrates into caproic acid, along with other metabolic end-products (Kim et al. 2015). *Caproiciproducens* ASV3 had a 100.0% BLAST similarity to *Thermocaproicibacter melissae* (Table S4), a thermophilic chain-elongating bacterium isolated from a thermophilic fermenter (Van Nguyen et al. 2023).

These compositional patterns were reflected in overall microbial diversity. Alpha-diversity analysis indicated a trend toward higher Shannon diversity at elevated carbohydrate concentrations, consistent with the increasing abundance of *Bacillaceae* genera (Table S5). Beta-diversity analysis using PCoA based on Bray-Curtis dissimilarity (Fig. S8) suggested that community composition was influenced by both substrate type and concentration. Samples from glucose- and xylose-fed reactors tended to separate along the first principal coordinate (32.3% of variance), while a gradual trend along the second coordinate (24.9% of variance) reflected differences in substrate concentration, with samples from higher concentrations tending toward one end of the axis.

## Discussion

### Glucose concentration influences the selection of metabolic pathways leading to lactic acid or a mixture of volatile fatty acids

This study demonstrates how glucose concentration in the reactor influences carboxylic acid production in thermophilic mixed-culture fermentation. Higher substrate concentrations favored lactic acid production over a mixture of VFAs, whereas VFAs predominated when glucose concentration in the reactor remained consistently close to 0 mg COD·L^−1^ (Fig. 1).

Lactic acid represented 76 ± 2% of the soluble fermentation products at the higher initial glucose concentration tested (610 mg COD·L^−1^) (Fig. 1B). This high proportion of lactic acid suggests that homolactic fermentation was the predominant metabolic pathway. In this metabolic pathway, one molecule of glucose is converted into two molecules of lactic acid (Table 4). Alternative lactic acid production pathways, such as heterolactic fermentation, cannot be excluded as minor metabolic routes, since fermentation products such as acetic acid and ethanol were also found in the product spectrum (Spormann 2023).

**Table 4:** Main metabolic fermentation reactions and theoretical ATP yield per mole of glucose or xylose. ATP yields are theoretical estimates based on the primary metabolic pathways involved. For homolactic fermentation, the ATP yield corresponds to the net ATP produced through glycolysis. For acetate-butyrate fermentation, ATP yields were estimated from glycolytic ATP production combined with acetyl-CoA dependent pathways that result in the formation of acetate and butyrate (Regueira et al. 2018; Spormann 2023; Temudo et al. 2007)

Metabolic pathway	Conversion reaction	Theoretical ATP yield (mol ATP·mol^−1^)
Homolactic fermentation	Glucose → 2 Lactate^−^ + 2 H^+^	2.00
	Xylose → 1.67 Lactate^−^ + 1.67 H^+^	1.67
Acetate-butyrate fermentation	Glucose + 2.67 H_2_O → 0.67 Acetate^−^ + 0.67 Butyrate^−^ + 2.67 H_2_ + 2 HCO_3_^−^ + 3.33 H^+^	3.33
	Xylose + 2.22 H_2_O → 0.56 Acetate^−^ + 0.56 Butyrate^−^ + 2.22 H_2_ + 1.67 HCO_3_^−^ + 2.78 H^+^	2.78

The production of lactic acid decreased as the initial concentration of glucose in the reactor was reduced, while the selectivity toward a mixture of VFAs increased (Fig. 1). This trend was observed across both reactors R1 and R2. At the lowest glucose concentration tested (0 mg COD·L^−1^), VFAs represented 83–97% of the total soluble fermentation products. Butyric acid was the main VFA produced, followed by acetic acid. Both acids are common fermentation products of glucose metabolism under mesophilic and thermophilic conditions (Temudo et al. 2007; Zhang et al. 2015), although the relative proportions of each metabolite can vary depending on the experimental conditions. Several studies have reported butyric acid as the main product under thermophilic conditions at mildly acidic pH (Arras et al. 2019; Zhang et al. 2015). This elevated proportion of butyric acid is consistent with the high H_2_ proportion in the headspace, typical of thermophilic and acidic environments. A high H_2_ partial pressure favors the formation of more reduced acids like butyric acid over more oxidized products such as acetic acid (Spormann 2023) and inhibits the oxidation of longer-chain acids to acetate and H_2_, favoring butyric acid accumulation (Müller et al. 2010). Additionally, butyric acid may also be produced from lactate oxidation, or via chain elongation using various electron donors such as sugars, lactic acid, or ethanol (Sakarika et al. 2023; Spirito et al. 2014). To date, ethanol-based chain elongation has not been reported at thermophilic conditions. Butyrate formation from lactate oxidation or via chain elongation using sugars or lactate as electron donors also appears unlikely when considering the observed product molar ratio. In reactor R2, the molar ratio of acetic acid to butyric acid was approximately 0.66:0.69 per 1 mol of glucose consumed under continuous feeding (at 0 mg COD·L^−1^). This product ratio is similar to that reported for acetate-butyrate fermentation when considering the electron bifurcation pathway, in which a molar ratio of 0.67:0.67 of acetic to butyric acid is expected per each mole of glucose (Regueira et al. 2018). This suggests that the acetate-butyrate pathway is the most likely metabolic route.

This acetic to butyric acid molar ratio was not maintained in reactor R1 due to the formation of other metabolites such as ethanol. Reactor R1 was fed with an influent containing higher glucose concentrations (18.2 g COD·L^−1^) than reactor R2 (7.8 g COD·L^−1^). An increase in ethanol yield at high influent substrate concentrations was also reported by Temudo et al. (2008), who observed higher ethanol formation when the concentration of glucose or xylose in the influent was increased from 4 to 25 g·L^−1^ (30 °C and pH 8). The production of ethanol under these conditions can be attributed to the higher substrate flux, which leads to an excess of reducing equivalents (e.g., NADH) during glucose metabolism. Under high H_2_ partial pressure, the reoxidation of NADH to produce H_2_ becomes thermodynamically unfavorable. To maintain redox balance, the system shifts toward the formation of more reduced compounds like ethanol (Temudo et al. 2007).

Similar shifts in product spectrum from lactic acid to a mixture of VFAs in response to glucose concentrations have been previously observed in mixed-culture fermentations. Zhang et al. (2015) reported that thermophilic glucose fermentation favored VFA production when glucose was fully consumed, whereas lactic acid represented 26% of the fermentation products when the organic loading rate was increased and glucose was not completely consumed (8 g COD·L^−1^ of remaining glucose in the reactor). Similar results were obtained by Hoelzle et al. (2021) at mesophilic temperatures. A mixture of VFAs was obtained when substrate was depleted, while steady lactic acid production (around 40% selectivity) was achieved when glucose was not completely consumed (1.5 to 6.1 g COD·L^−1^ of remaining glucose in the reactor). In both studies, the accumulated glucose concentration in the reactor was much higher than the maximum concentration tested in the present work (610 mg COD·L^−1^). Yet, the corresponding lactic acid selectivity was lower in comparison to the one obtained in this study (76 ± 2%). Although the exact cause of these differences remains unknown, they might be related to the different operational conditions applied. In both studies, the experiments were performed in CSTRs, and substrate concentration was modulated by varying the glucose concentration in the influent (Hoelzle et al. 2021; Zhang et al. 2015). The higher concentrations tested could have led to VFA inhibition or toxicity (Siegert and Banks 2005), which may explain why glucose was not fully consumed. Conversely, in the present study, substrate concentration in the reactor was adjusted by modifying the operational mode from SBR to continuous operation, without changing the concentration of glucose in the influent. Toxic or inhibitory VFA concentrations were never reached as substrate was always fully consumed. These differences may partly explain the observed discrepancies in bulk glucose concentration and lactic acid production between the studies. Other studies using complex substrates have also reported a shift from a mixture of VFAs toward lactic acid at increased substrate availability. During thermophilic fermentation of food waste, VFAs were the main product under continuous operation, whereas lactic acid became the primary product under SBR operation with 48–72 h cycles and a volume exchange ratio of 20% (Vulart et al. 2025). Under these conditions, lactic acid selectivity reached up to 90–95%, highlighting the potential to achieve a higher proportion of lactic acid under conditions of increased substrate concentrations. Accordingly, Liu et al. (2023) reported an increased lactic acid-to-VFAs ratio in an SBR when higher volume exchange ratios and shorter cycle times were applied.

From an energetic perspective, lactic acid production from glucose is less favorable than acetate-butyrate fermentation (Table 4). Lactic acid fermentation generates 2 mol ATP per 1 mol of glucose, while acetate-butyrate fermentation yields approximately 3.33 mol ATP (Regueira et al. 2018). Despite the lower ATP yield, it has been shown that microorganisms favor lactic acid production under high substrate concentrations, which has been recently explained by the resource allocation theory (Regueira et al. 2021a, b). This theory suggests that cellular processes such as catabolism, biosynthesis, and transport compete for a limited pool of proteins. Under low substrate availability, the proteome is not a limiting factor, and microorganisms can favor metabolic pathways with higher ATP yields, like acetate-butyrate fermentation. In contrast, under high substrate availability, microorganisms tend to favor pathways with greater proteome efficiency, even if this yields less ATP. Therefore, the lactic acid production observed in this study at elevated substrate concentrations likely represents a proteome-efficient strategy despite the lower ATP yield, allowing the cells to rapidly process the glucose available.

### The shift from lactic acid to volatile fatty acid production depends on the type of carbohydrate

Xylose is one of the most abundant monomeric sugars in lignocellulosic materials, and it is considered a model carbohydrate monomer for pentoses (Narisetty et al. 2022). This work showed that a similar shift in product formation from lactic acid to VFAs took place when using xylose as substrate (Fig. 2). At high xylose concentrations in the reactor (670 mg COD·L^−1^), lactic acid was the main product, accounting for 68 ± 2% of the total soluble fermentation products. This high proportion of lactic acid suggests that homolactic fermentation was likely the predominant metabolic pathway under conditions of high xylose concentrations, as was also observed with glucose. Few microbial species have been shown to convert pentoses to lactic acid via homolactic fermentation, including *H. coagulans* (Glaser and Venus 2017; Ou et al. 2011; Patel et al. 2006), which had been identified as ASV11 in the xylose-fed reactor, with a BLAST similarity of 100.0% (Table S4, Fig. S7). This species mainly metabolizes xylose via the pentose phosphate pathway (PPP), converting three molecules of xylose into five of lactic acid (Table 4) (Patel et al. 2006). *Thermoanaerobacterium* species can also metabolize xylose to lactic acid, although they typically produce acetic acid, butyric acid, and/or ethanol as co-products (Lee et al. 1993). Although lactic acid production has been widely observed in fermentations involving complex organic matter (e.g., food waste or lignocellulosic biomass) (Liu et al. 2023; Tashiro et al. 2013; Vulart et al. 2025; Yang et al. 2022), to the authors’ knowledge, no studies to date report lactic acid as the main product from xylose fermentation using mixed cultures.

As in the glucose-fed systems, low xylose concentrations in the reactor (0 mg COD·L^−1^) led to a mixture of VFAs, which represented 91 ± 1% of the soluble fermentation products (Fig. 2). Acetic and butyric acid were the main fermentation products, with lower amounts of ethanol also present in the reactor. This suggests acetate-butyrate fermentation was the main metabolic pathway. The product profile is also similar to that reported by Iglesias-Riobó et al. (2024) for xylose fermentation under continuous operation at 37 °C and pH 5.

Despite the similarities with the glucose-fed reactors at both higher and lower substrate concentrations, the product distribution observed when using xylose as carbon source differed from that of the glucose-fed reactors under comparable intermediate substrate concentrations (Figs. 1, 2). In the glucose-fed reactors R1 and R2, lactic acid was still present in the product spectrum with initial glucose concentrations of 100–240 mg COD·L^−1^. Conversely, with similar initial xylose concentrations in reactor R3 (270 mg COD·L^−1^), VFAs were the main product, but no lactic acid was observed in the final product spectrum. The mix of VFAs included acetic, butyric, and caproic acid. In contrast to other experimental conditions, the microbial community in this period was dominated by the genus *Caproiciproducens*, which includes several species that produce caproic acid (Kim et al. 2015). BLAST analysis identified *Caproiciproducens* ASV3 as *T. melissae* with a similarity of 100.0%. Caproic acid production from xylose has been previously reported at both mesophilic (Qian et al. 2020; Tang et al. 2022) and thermophilic conditions (Sakarika et al. 2023). Caproic acid can be produced via several chain elongation pathways, depending on the electron donor (sugars, lactic acid, or ethanol). To date, only sugar-chain elongation to caproic acid has been reported at thermophilic conditions (Sakarika et al. 2023; Van Nguyen et al. 2023), indicating that xylose is likely the electron donor for caproic acid production. Accordingly, *T. melissae* is a thermophilic sugar-based chain elongator able to produce caproic acid from xylose (Van Nguyen et al. 2023). The reason why caproic acid is only produced under this specific experimental condition remains unclear.

Therefore, the product profile of the xylose-fed reactor at intermediate initial xylose concentrations stands in clear contrast to that of the glucose-fed reactors, where caproic acid was not produced and lactic acid remained a significant product under similar operation conditions. These differences highlight that product selectivity depends not only on substrate concentrations but also on the type of carbohydrate. Glucose and xylose are metabolized via different metabolic pathways, with different energetic efficiencies (Temudo et al. 2007, 2009). In *Bacillus* and *Thermoanaerobacterium* species, glucose and other hexoses are primarily catabolized via the Embden-Meyerhof-Parnas (EMP) pathway, whereas pentoses such as xylose are usually metabolized through the non-oxidative PPP (Chen et al. 2020; Jacobson et al. 2020; Stülke and Hillen 2000; Wushensky et al. 2018). Glucose catabolism via the EMP pathway yields 2 net ATP and 2 NADH per glucose. Xylose catabolism through the non-oxidative PPP generates no direct ATP, but the resulting intermediates produce around 1.67 ATP and 1.67 NADH per xylose when metabolized via the EMP pathway (Spormann 2023). Glucose and xylose are also taken up via different mechanisms that differ in their energy requirements. For instance, several types of transporters have been identified in Firmicutes for xylose uptake (Gu et al. 2010). Major facilitator superfamily-type xylose transporters, orthologous to XylT (first described in *Bacillus megaterium*), have been identified in several species of *Bacilli* and *Clostridia*. These transporters function as xylose/proton symporters, without direct ATP consumption. ABC-type xylose transporters, orthologous to XylFGH originally described in *Escherichia coli*, have also been found in species of *Clostridiales*, *Thermoanaerobacterales*, and *Bacillales*, including *T. thermosaccharolyticum* (Zhao et al. 2020). These transporters are ATP-dependent and consume one molecule of ATP per molecule of xylose transported (Jeckelmann and Erni 2020). Conversely, glucose is usually taken up via phosphotransferase systems or facilitated diffusion, often with no net ATP cost, or with a more energetically favorable mechanism such as proton symporters (Jahreis et al. 2008). Given that lower ATP yields are obtained from xylose catabolism, it is likely that production shifts toward the more energy-efficient acetate-butyrate fermentation over lactic acid fermentation when xylose is the primary carbon source (Table 4).

### Changes in product profile stem from a metabolic shift within the dominant species and variations in microbial community composition

The shift in fermentation products, favoring lactic acid at high substrate concentrations and VFAs at low concentrations, was observed across all three reactors, regardless of the carbon source used (Figs. 1, 2). The microbial community composition was also similar in all three systems, dominated by *Thermoanaerobacterium* and genera within the *Bacillaceae* family (Fig. 3). The latter were more abundant under conditions characterized by intermittent but higher substrate concentrations, whereas a continuous but lower substrate supply promoted the enrichment of *Thermoanaerobacterium*. This microbial community composition is consistent with the observed product spectrum. Several species within the *Bacillaceae* family are well-known for their ability to produce lactic acid, including *H. coagulans* (Payot et al. 1999), *Bacillus licheniformis* (Wang et al. 2011), and *Bacillus subtilis* (Tongpim et al. 2014). In contrast, *Thermoanaerobacterium* species can produce a mixture of lactic acid, acetic acid, butyric acid, and/or ethanol (Lee et al. 1993).

Despite the selective pressure on the microbial community imposed by substrate concentrations, influencing the relative abundance of specific genera, both *Thermoanaerobacterium* and members of the *Bacillaceae* family coexisted within a certain range of substrate concentrations (Fig. 3). Previous studies have also reported the simultaneous presence of the genera *Thermoanaerobacterium* and *Bacillus* in thermophilic reactors where the fermentation profile was dominated by lactic acid, with minimal accumulation of acetic acid, butyric acid, and ethanol (Yang et al. 2022). These observations raise the question of whether the product spectrum observed was primarily the result of a metabolic shift within the dominant microbial species or a consequence of changes in community composition. Despite the increase in the relative abundance of genera within the *Bacillaceae* family at higher substrate concentrations, *Thermoanaerobacterium* remained the dominant genus in all reactors. Given its ability to produce both lactic acid and VFAs (such as acetic and butyric acid) (Lee et al. 1993), the observed product shifts could be the result of a redistribution of the electron flow through different metabolic pathways, depending on substrate concentrations. This behavior has been previously observed in other species. For example, *Lactococcus lactis* can modulate its metabolism depending on carbohydrate concentrations. In the presence of high substrate concentrations, this microorganism rapidly metabolizes glucose to lactic acid via homolactic fermentation. Conversely, under low substrate conditions, *L. lactis* shifts to a slower but more energy-efficient pathway that produces acetic acid and ethanol, resulting in the formation of one additional mole ATP per mole of glucose (Spormann 2023). The presence of different *Thermoanaerobacterium* ASVs could also partially explain the shift in the product spectrum. Based on BLAST results, some of these ASVs showed similarity to different *Thermoanaerobacterium* species. Several ASVs also matched *T. thermosaccharolyticum*, suggesting the possible coexistence of distinct strains of this species within the reactors. Such inter- and intraspecific diversity could contribute to variations in metabolic activity and substrate utilization. Van Nguyen (2023) reported strain-dependent variations in carbohydrate utilization by *T. thermosaccharolyticum*, resulting in differences in their fermentation products. However, no clear correlation was observed between the dominant ASVs and substrate concentration in the reactor (Fig. S7).

Taken together, these findings suggest that the observed shifts in product spectrum likely stem from a combination of both a metabolic shift within the dominant species and changes in the microbial community composition. It is important to note that this experiment was conducted at relatively low substrate concentrations. A greater increase in substrate concentrations could lead to a shift in the dominant genera within the microbial community. It has been reported that, in thermophilic fermentation of food waste, genera within the *Bacillaceae* family dominated the reactor operated as sequencing batch with a volume exchange ratio of 20% and cycle time of 48–72 h, while *Caproiciproducens* dominated the reactor under continuous operation (Vulart et al. 2025). Although the complexity of food waste makes it difficult to determine the exact concentration of monomers in these reactors, the amount of volume replaced under SBR operation likely contained higher concentrations of carbohydrates than those tested in this study, suggesting that higher substrate concentrations could drive changes in the microbial community composition.

## Conclusions

This study shows that carbohydrate concentration plays a key role in determining product formation in thermophilic mixed-culture fermentations. High carbohydrate concentrations (650–670 mg COD·L^−1^) favored lactic acid production, while lower substrate concentrations (0 mg COD·L^−1^) led to the formation of a mixture of VFAs. Despite this general trend, the concentration at which this shift from lactic acid to VFAs was observed depended on the type of carbohydrate. Glucose and xylose are metabolized via different metabolic pathways, with xylose metabolism being less energetically efficient than glucose metabolism. Given that the ATP yield from lactic acid fermentation is lower than that from acetate-butyrate fermentation, microorganisms likely shifted toward the more energy-efficient acetate-butyrate fermentation when xylose was the primary carbon source to maximize the energy yield. Microbial community analyses revealed that *Thermoanaerobacterium* dominated across most of the tested conditions, while members within the *Bacillaceae* family increased in relative abundance under high carbohydrate concentrations. These results suggest that the shifts in product distribution were driven by both metabolic activity changes within dominant microorganisms and modifications in the microbial community structure. Overall, these findings highlight the critical role of substrate concentrations in determining the fermentation product spectrum, providing valuable insights for directing bioprocesses toward the targeted production of specific carboxylic acids.

## Supplementary Information

Below is the link to the electronic supplementary material.

ESM 1 (PDF 1.52 MB)

## Data Availability

The raw fastq files that served as a basis for the microbial community analysis were deposited in the National Center for Biotechnology Information (NCBI) database (accession number PRJNA1347372). All other data can be made available upon request.
