# Supplementary material for: Carbohydrate concentration and type drive product selectivity to a mixture of volatile fatty acids or lactic acid in thermophilic mixed-culture fermentation
Source: Appl Microbiol Biotechnol. 2026 Apr 11;110:143. doi: 10.1007/s00253-026-13806-0 (PMC13180770; doi:10.1007/s00253-026-13806-0)
Supplement: Supplementary file 1 [file 253_2026_13806_MOESM1_ESM.pdf]

Supplementary information to: Carbohydrate concentration and type drive product selectivity to a mixture of volatile fatty acids or lactic acid in thermophilic mixed-culture fermentation

Laia Vulart[a,b,c], Néstor Izcara[b,c], Ángel Estévez[b,c], Enrique Peiro[a], Francesc Gòdia[a], Ramon Ganigué[b,c]

[a]Departament d'Enginyeria Química, Biològica i Ambiental, Universitat Autònoma de Barcelona, 08193 Bellaterra (Cerdanyola del Vallès), Spain

[b]Center for Microbial Ecology and Technology (CMET), Ghent University, 9052 Ghent, Belgium

[c]Centre for Advanced Process Technology for Urban Resource Recovery (CAPTURE), 9052 Ghent, Belgium

Corresponding author:

Laia Vulart

Universitat Autònoma de Barcelona, Escola d'Enginyeria, Departament d'Enginyeria Química, Biològica i Ambiental, 08193 Bellaterra (Cerdanyola del Vallès), Spain

Email: laia.vulart@uab.cat

Supplementary figures

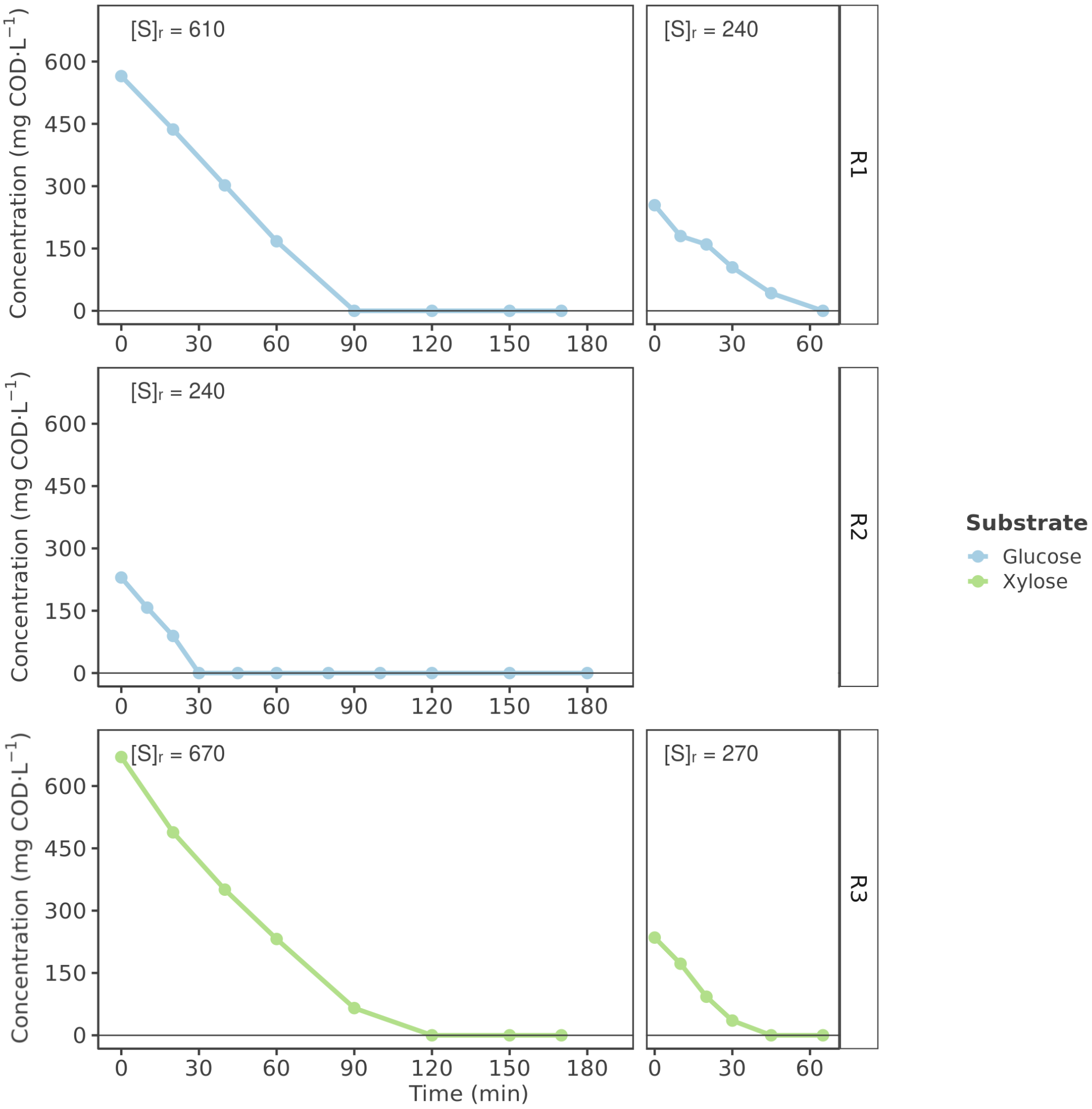

Fig. S1 Substrate concentration (mg $COD \cdot L^{-1}$) over time during the 3.0 h and 1.2 h sequencing batch cycles in reactors R1, R2 and R3. Reactor R1, R2 and R3 were fed with an influent containing 18.2 g $COD \cdot L^{-1}$ of glucose, 7.8 g $COD \cdot L^{-1}$ of glucose, and 21.4 g $COD \cdot L^{-1}$ of xylose, respectively

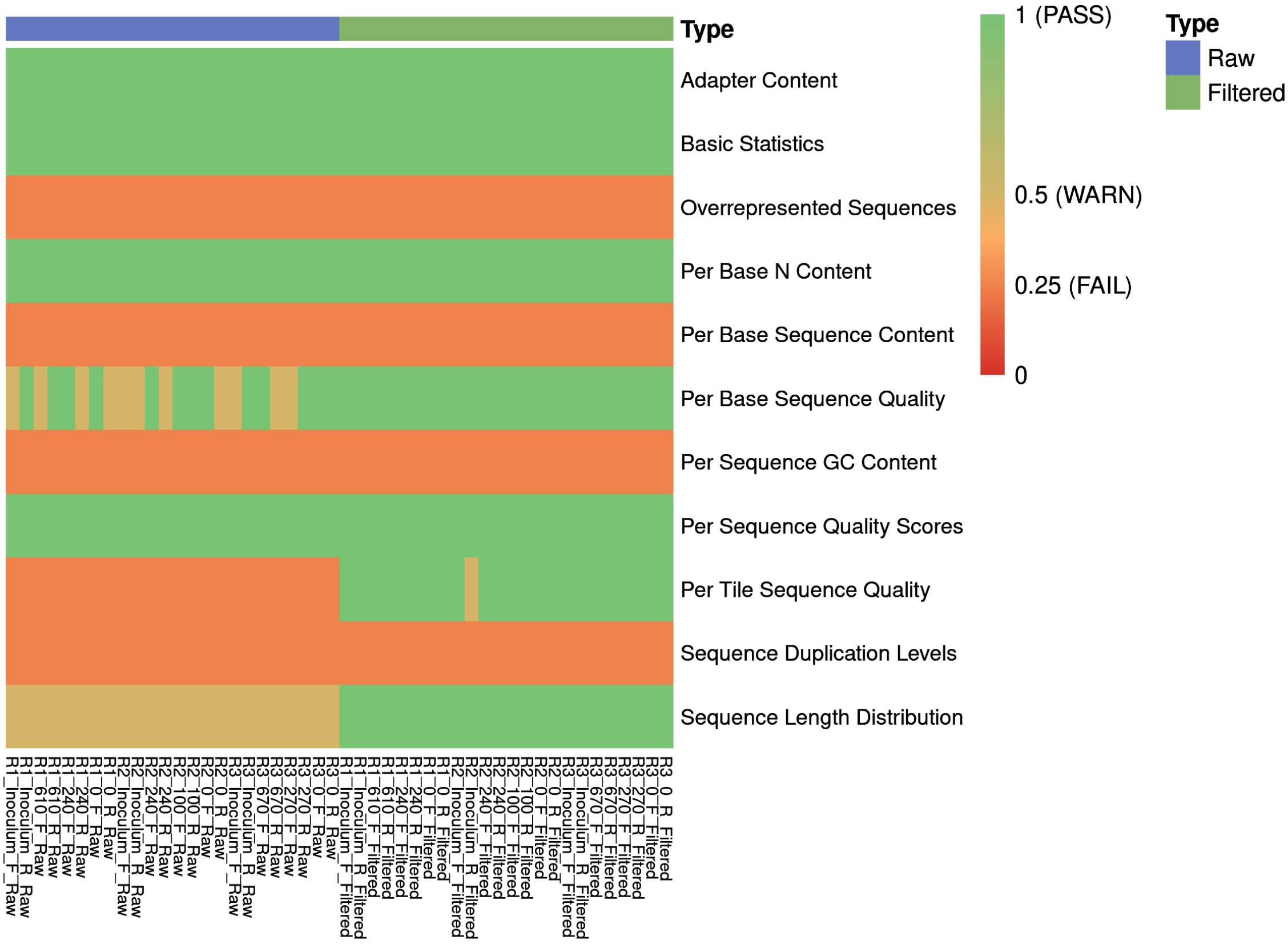

Fig. S2 FastQC quality-control summary for all raw and filtered 16S rRNA amplicon sequencing samples, generated using MultiQC (MultiQC | Seqera, n.d.)

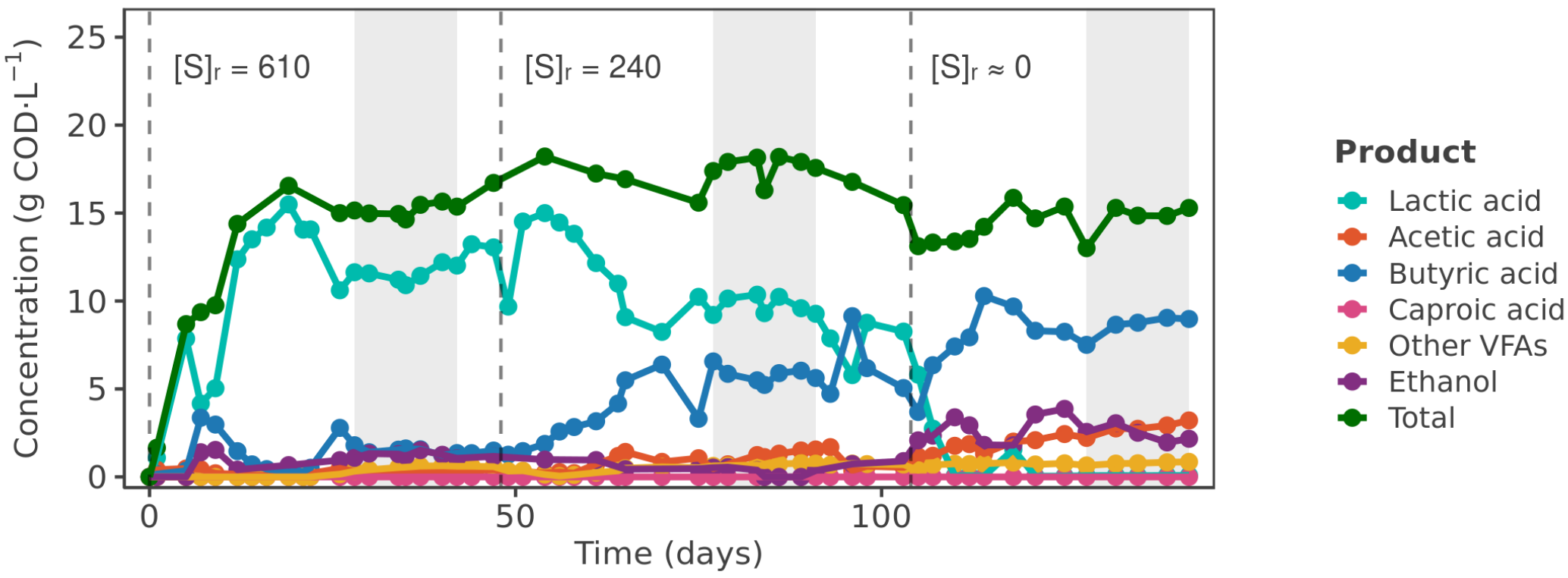

Fig. S3 Product profile (mg COD·L^{-1}) over time of the soluble fermentation products in reactor R1, fed with 18.2 g·L^{-1} of glucose. The tested reactor glucose concentrations ([S]$_r$) were 610 mg COD·L^{-1}, 240 mg COD·L^{-1}, and 0 mg COD·L^{-1}. Grey-shaded areas represent the periods of steady-state performance. "Other VFAs" include propionic acid, isobutyric acid, valeric acid, isovaleric acid, and isocaproic acid

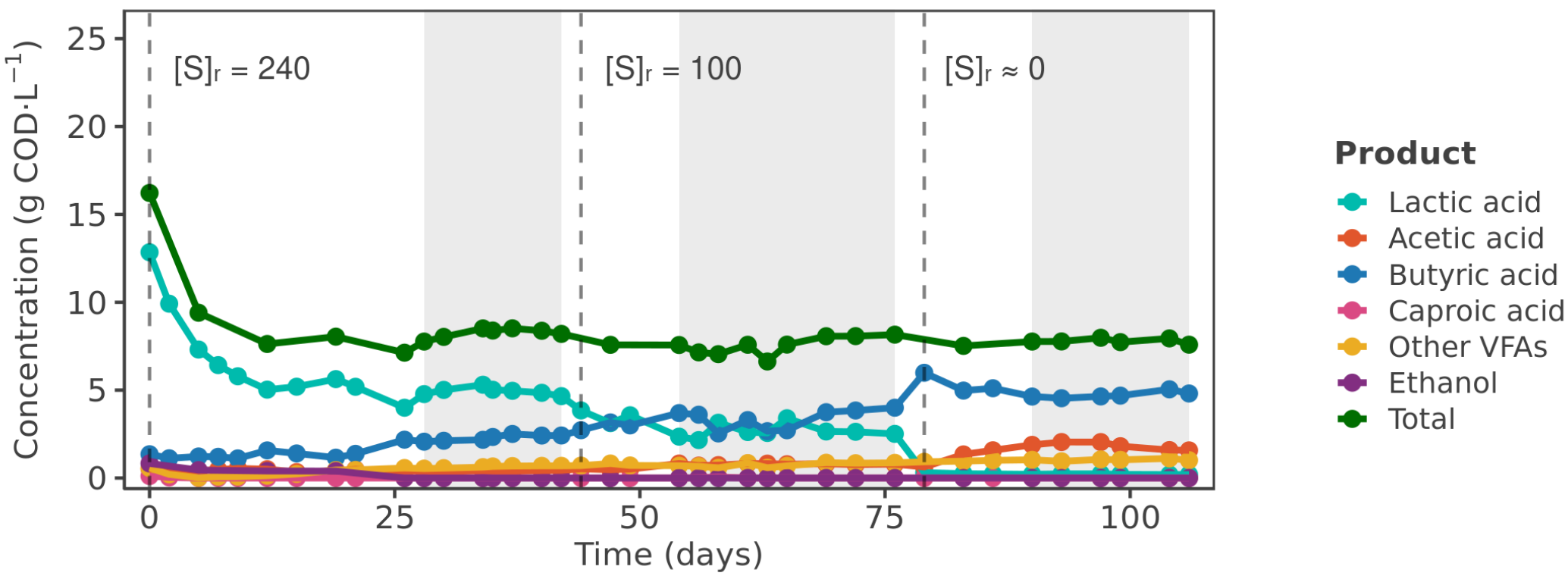

Fig. S4 Product profile (mg COD·L^{-1}) over time of the soluble fermentation products in reactor R2, fed with 7.8 g·L^{-1} of glucose. The tested reactor glucose concentrations ([S]$_r$) were 240 mg COD·L^{-1}, 100 mg COD·L^{-1}, and 0 mg COD·L^{-1}. Grey-shaded areas represent the periods of steady-state performance. "Other VFAs" include propionic acid, isobutyric acid, valeric acid, isovaleric acid, and isocaproic acid

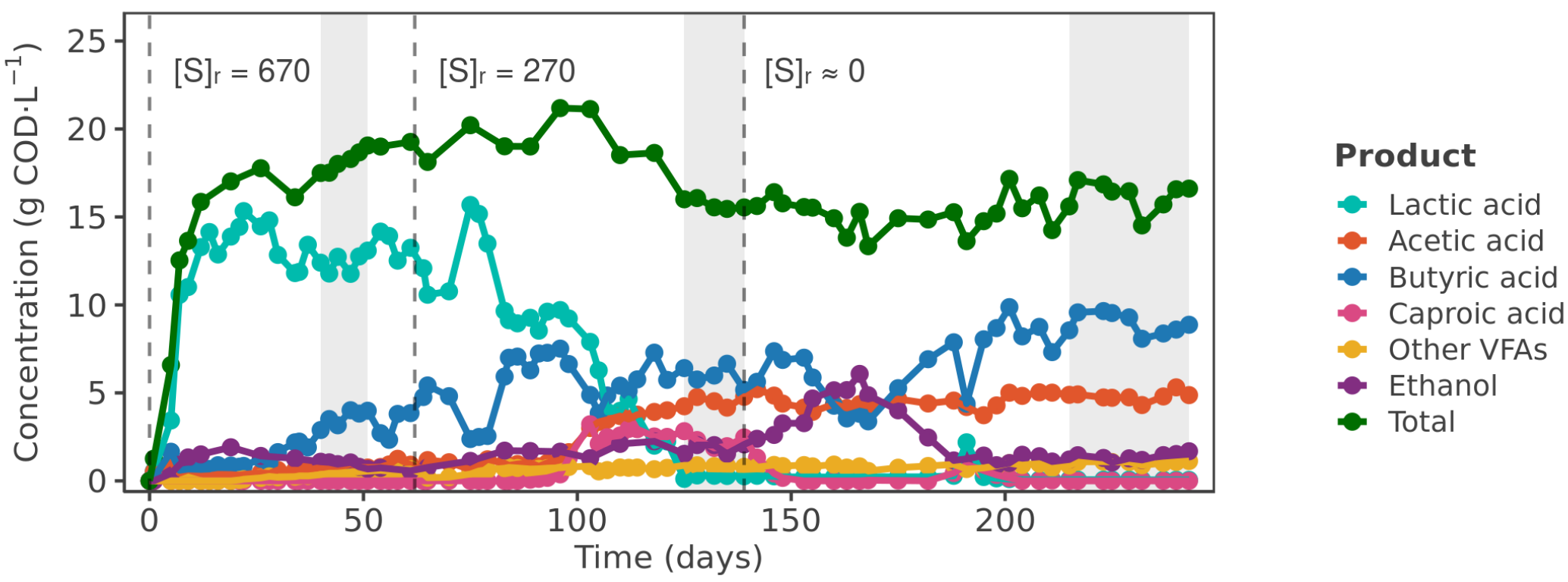

Fig. S5 Product profile (g COD·L^{-1}) over time of the soluble fermentation products in reactor R3, fed with 21.4 g·L^{-1} of xylose. The tested reactor xylose concentrations ([S]$_r$) were 670 mg COD·L^{-1}, 270 mg COD·L^{-1}, and 0 mg COD·L^{-1}. Grey-shaded areas represent the periods of steady-state performance. "Other VFAs" include propionic acid, isobutyric acid, valeric acid, isovaleric acid, and isocaproic acid

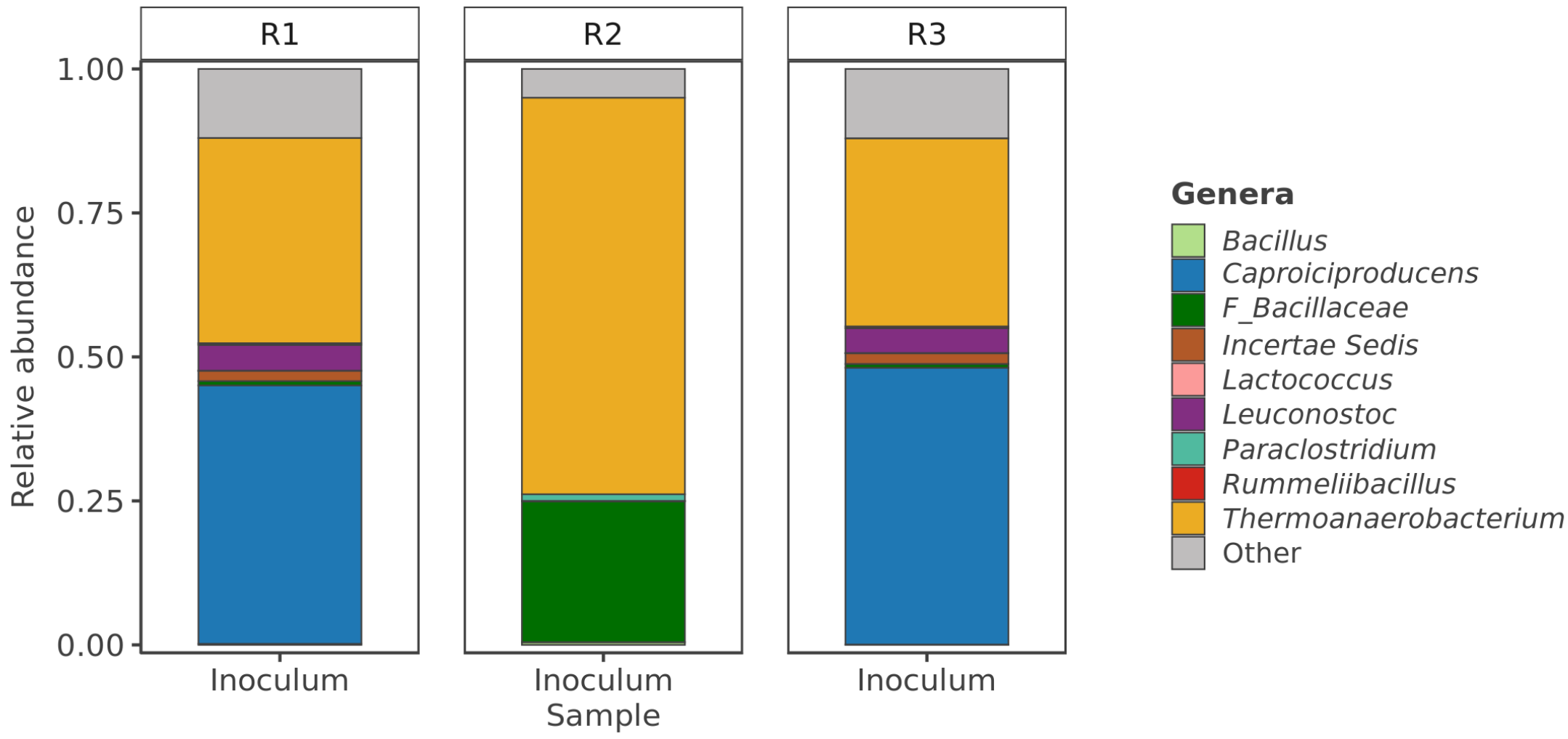

Fig. S6 Relative abundance of bacteria at the genus level in the inoculum of R1, R2 and R3, fed with 18.2 g COD·L^{-1} of glucose, 7.8 g COD·L^{-1} of glucose, and 21.4 g COD·L^{-1} of xylose, respectively

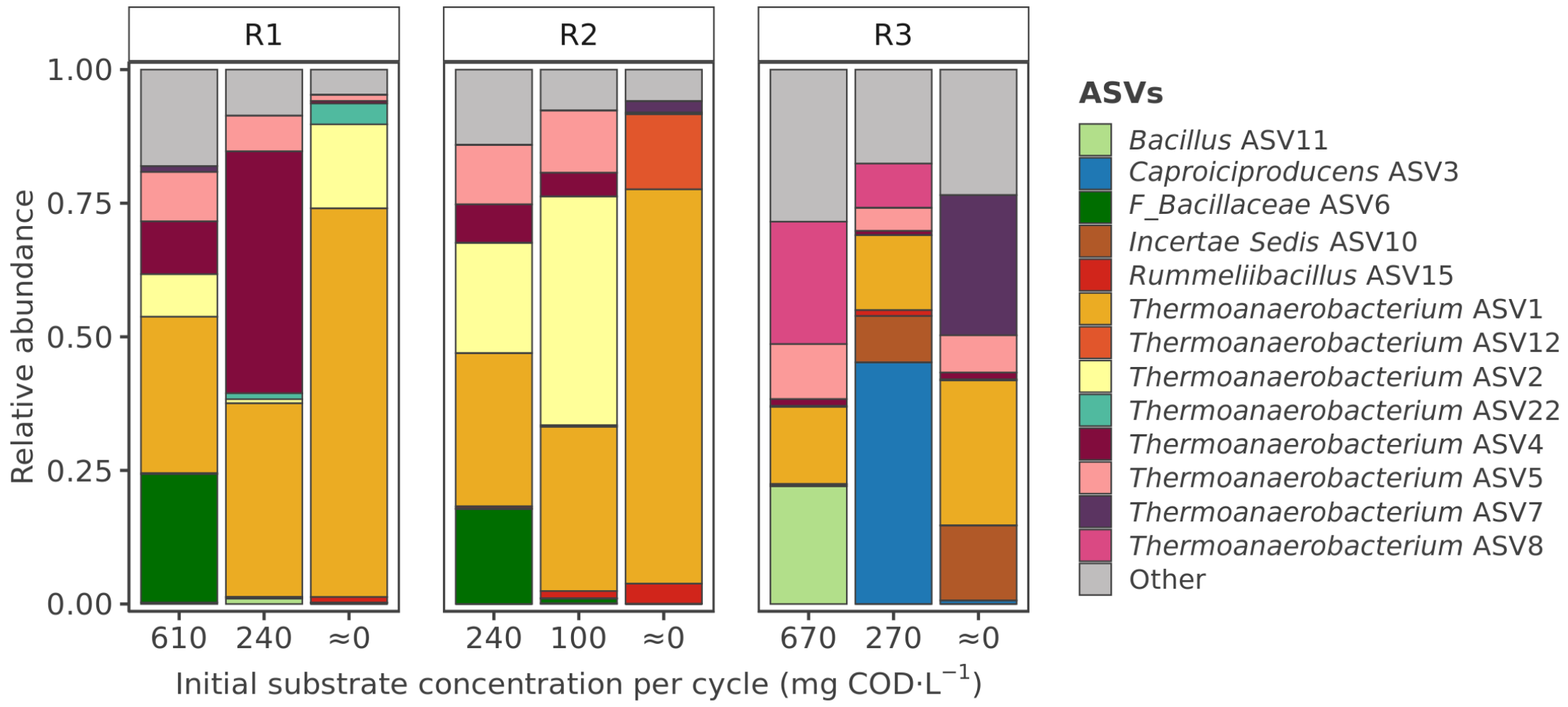

Fig. S7 Relative abundance of bacteria at the amplicon sequence variant level in reactors R1, R2 and R3, fed with 18.2 g COD·L^{-1} of glucose, 7.8 g COD·L^{-1} of glucose, and 21.4 g COD·L^{-1} of xylose, respectively, at different reactor substrate concentrations. For reactor R1, samples 610, 240, and ≈0 correspond to days 42, 91, and 142, respectively. For reactor R2, these correspond to days 42, 76, and 106, and for reactor R3 to days 51, 139, and 243, respectively

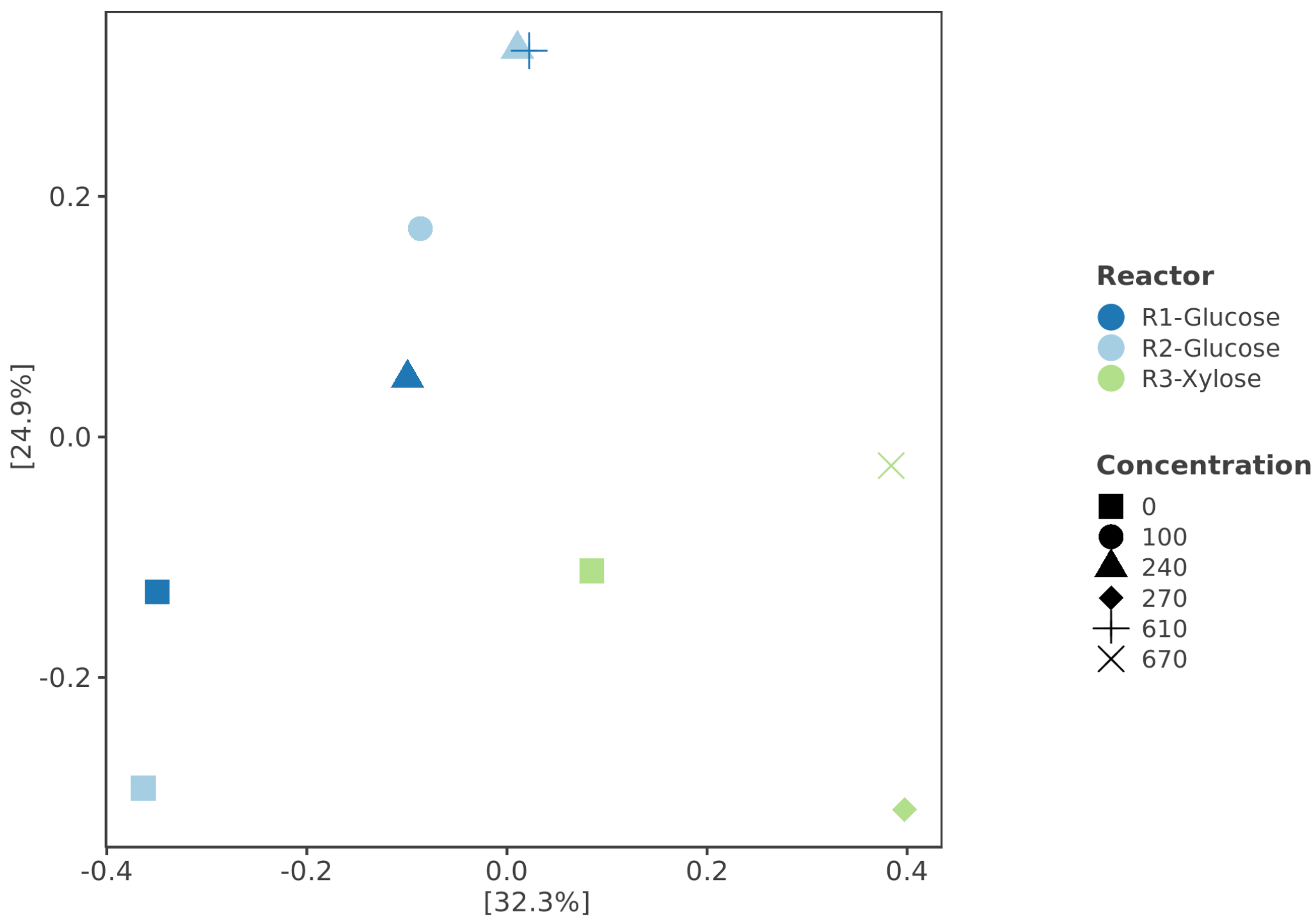

Fig. S8 Principal Coordinates Analysis of Bray-Curtis dissimilarities showing differences in microbial community composition across reactors and substrate concentrations. Reactors R1, R2 and R3 were fed with 18.2 g COD·L^{-1} of glucose, 7.8 g COD·L^{-1} of glucose, and 21.4 g COD·L^{-1} of xylose, respectively. For reactor R1, samples 610, 240, and ≈0 correspond to days 42, 91, and 142, respectively. For reactor R2, these correspond to days 42, 76, and 106, and for reactor R3 to days 51, 139, and 243, respectively

Supplementary tables

Table S1 Medium composition

Medium		
**Compound**	**Concentration**	**Unit**
$MgCl_2 \cdot 6H_2O$	1.00	$g \cdot L^{-1}$
$CaCl_2 \cdot 2H_2O$	0.50	$g \cdot L^{-1}$
$NaH_2PO_4 \cdot 2H_2O$	0.50	$g \cdot L^{-1}$
Na_2SO_4	0.10	$g \cdot L^{-1}$
KCl	1.00	$g \cdot L^{-1}$
NH_4Cl	2.00	$g \cdot L^{-1}$
Yeast extract	1.00	$g \cdot L^{-1}$
Tryptone	4.00	$g \cdot L^{-1}$
Trace elements SL10 (10x)	1.00	$mL \cdot L^{-1}$
Selenite & Tungstate Se-W (10x)	1.00	$mL \cdot L^{-1}$
7-Vitamin solution (10x)	0.10	$mL \cdot L^{-1}$

Table S2 Trace elements SL10, Selenite & Tungstate Se-W and 7-Vitamin solutions

Trace elements SL10 (10x)		
**Compound**	**Concentration**	**Unit**
HCl	28.07	$g \cdot L^{-1}$
$FeCl_2 \cdot 4H_2O$	15.00	$g \cdot L^{-1}$
$ZnCl_2$	0.70	$g \cdot L^{-1}$
$MnCl_2 \cdot 4H_2O$	1.00	$g \cdot L^{-1}$
H_3BO_3	0.06	$g \cdot L^{-1}$
$CoCl_2 \cdot 6H_2O$	1.90	$g \cdot L^{-1}$
$CuCl_2 \cdot 2H_2O$	0.02	$g \cdot L^{-1}$
$NiCl_2 \cdot 6H_2O$	0.24	$g \cdot L^{-1}$
$Na_2MoO_4 \cdot 2H_2O$	0.36	$g \cdot L^{-1}$
**Selenite and Tungstate Se-W (10x)**		
**Compound**	**Concentration**	**Unit**
NaOH	5.00	$g \cdot L^{-1}$
$Na_2SeO_3 \cdot 5H_2O$	30.00	$g \cdot L^{-1}$
$Na_2WO_4 \cdot 2H_2O$	0.04	$g \cdot L^{-1}$
**7-Vitamin solution (10x)**		
**Compound**	**Concentration**	**Unit**
Vitamin B12	1.00	$g \cdot L^{-1}$
p-Aminobenzoic acid	0.80	$g \cdot L^{-1}$
D(+) Biotin	0.20	$g \cdot L^{-1}$
Nicotinic acid	2.00	$g \cdot L^{-1}$
Calcium pantothenate	1.00	$g \cdot L^{-1}$
Pyridoxine hydrochloride	3.00	$g \cdot L^{-1}$
Thiamine-HCl $\cdot$ $2H_2O$	2.00	$g \cdot L^{-1}$

Table S3 Read-tracking summary showing the number of sequencing reads retained at each step of the processing pipeline

Sample	Input	Filtered	DenoisedF	DenoisedR	Merged	Non chimera	ASV filtered
**R1_Inoculum**	36856	26416	26274	26197	25526	24283	24283
**R1_610**	56341	40740	40617	40697	39961	34853	34853
**R1_240**	62372	45993	45913	45926	45239	42436	42436
**R1_0**	63748	48266	48012	48159	47622	46048	46048
**R2_Inoculum**	36486	25316	25235	25202	24630	20905	20905
**R2_240**	60640	44119	44004	43996	43030	28440	28439
**R2_100**	59827	44840	44741	44720	43855	38937	38937
**R2_0**	67625	51190	51041	50991	50357	47562	47562
**R3_Inoculum**	31498	22456	22293	22337	21792	20844	20844
**R3_670**	73510	53380	53246	53316	52254	42651	42649
**R3_270**	53170	39597	39409	39512	38711	31145	31145
**R3_0**	79946	59568	59420	59416	58553	49799	49799

Table S4 Top five BLAST results for the most relevant amplicon sequence variants (ASVs) identified in the reactors, excluding uncultured/environmental sample sequences (consulted on 18-10-2025)

	Description	Identity (%)	Accession length
**ASV1**	*Thermoanaerobacterium thermosaccharolyticum* strain GD17 16S ribosomal RNA gene, partial sequence	100.00	1511
	Thermoanaerobacterium thermosaccharolyticum strain CT122 16S ribosomal RNA gene, partial sequence	100.00	1254
	Thermoanaerobacterium thermosaccharolyticum strain WC13 16S ribosomal RNA gene, partial sequence	100.00	1457
	Thermoanaerobacterium sp. MYST/2012-07 16S ribosomal RNA gene, partial sequence	100.00	1268
	Thermoanaerobacterium thermosaccharolyticum strain CTE78 16S ribosomal RNA gene, partial sequence	100.00	1322
**ASV2**	*Thermohydrogenium kirishiense* strain ZE-7 16S ribosomal RNA, partial sequence	100.00	1508
	Thermoanaerobacterium thermosaccharolyticum M0795, complete genome	100.00	2783395
	Thermoanaerobacterium thermosaccharolyticum strain GD17 16S ribosomal RNA gene, partial sequence	99.75	1511
	Thermoanaerobacterium thermosaccharolyticum strain CT122 16S ribosomal RNA gene, partial sequence	99.75	1254
	Thermoanaerobacterium thermosaccharolyticum strain WC13 16S ribosomal RNA gene, partial sequence	99.75	1457
**ASV3**	*Thermocaproicibacter melissae* strain MDTJ8 chromosome, complete genome	100.00	1941417
	Thermocaproicibacter melissae strain MDTJ8 16S ribosomal RNA, partial sequence	100.00	1516
	Oscillospiraceae bacterium strain MG12 16S ribosomal RNA gene, partial sequence	98.76	1320
	Oscillospiraceae bacterium strain MG13 16S ribosomal RNA gene, partial sequence	98.76	1397
	Oscillospiraceae bacterium strain HV4-5-B5C 16S ribosomal RNA gene, partial sequence	96.77	1360

**ASV4**	*Thermohydrogenium kirishiense* strain ZE-7 16S ribosomal RNA, partial sequence	99.26	1508
	Thermoanaerobacterium thermosaccharolyticum M0795, complete genome	99.26	2783395
	Thermoanaerobacterium thermosaccharolyticum strain GD17 16S ribosomal RNA gene, partial sequence	99.01	1511
	Thermoanaerobacterium thermosaccharolyticum strain CT122 16S ribosomal RNA gene, partial sequence	99.01	1254
	Thermoanaerobacterium thermosaccharolyticum strain WC13 16S ribosomal RNA gene, partial sequence	99.01	1457
**ASV5**	*Thermoanaerobacterium thermosaccharolyticum* strain D120-70 16S ribosomal RNA gene, complete sequence	100.00	1452
	Thermoanaerobacterium thermosaccharolyticum strain CT72 16S ribosomal RNA gene, partial sequence	100.00	733
	Thermoanaerobacterium thermosaccharolyticum strain CT6 16S ribosomal RNA gene, partial sequence	99.75	990
	Thermoanaerobacterium thermosaccharolyticum strain DJA2 16S ribosomal RNA gene, partial sequence	99.50	980
	Thermoanaerobacterium bryantii 16S ribosomal RNA gene, partial sequence	98.75	1329
**ASV7**	*Thermoanaerobacterium thermosaccharolyticum* strain F6 16S ribosomal RNA gene, partial sequence	100.00	1376
	Thermoanaerobacterium thermosaccharolyticum strain MJ1 chromosome, complete genome	99.75	2761367
	Thermoanaerobacterium thermosaccharolyticum strain GD17 16S ribosomal RNA gene, partial sequence	99.75	1511
	Thermoanaerobacterium thermosaccharolyticum strain CT122 16S ribosomal RNA gene, partial sequence	99.75	1254
	Thermoanaerobacterium thermosaccharolyticum strain WC13 16S ribosomal RNA gene, partial sequence	99.75	1457
**ASV8**	*Thermoanaerobacterium xylanolyticum* LX-11, complete genome	99.75	2534358
	Thermoanaerobacterium xylanolyticum strain LX-11 16S ribosomal RNA, partial sequence	99.75	1576
	Thermoanaerobacterium calidifontis strain Rx1 16S ribosomal RNA, partial sequence	99.75	1473
	Thermoanaerobacterium calidifontis JCM 18270 DNA, complete genome	99.75	2686457
	Thermoanaerobacterium thermosaccharolyticum gene for 16S ribosomal RNA, partial sequence, strain: JCA-5603	99.50	1283
**ASV11**	*Weizmannia coagulans* BKMTCR2-2 gene for 16S ribosomal RNA, partial sequence	100.00	1554
	Heyndrickxia coagulans strain V3984 16S ribosomal RNA gene, partial sequence	100.00	1354
	Heyndrickxia coagulans strain V1083 16S ribosomal RNA gene, partial sequence	100.00	1419
	Heyndrickxia coagulans strain V4093 16S ribosomal RNA gene, partial sequence	100.00	1369
	Heyndrickxia coagulans strain V1080 16S ribosomal RNA gene, partial sequence	100.00	1378
**ASV12**	*Thermoanaerobacterium xylanolyticum* LX-11, complete genome	100.00	2534358
	Thermoanaerobacterium xylanolyticum strain LX-11 16S ribosomal RNA, partial sequence	100.00	1576
	Thermoanaerobacterium calidifontis strain Rx1 16S ribosomal RNA, partial sequence	100.00	1473

	Thermoanaerobacterium calidifontis JCM 18270 DNA, complete genome	100.00	2686457
	Thermoanaerobacterium thermosaccharolyticum gene for 16S ribosomal RNA, partial sequence, strain: JCA-5603	99.75	1283
**ASV22**	*Thermoanaerobacterium thermosaccharolyticum* strain MJ1 chromosome, complete genome	100.00	2761367
	Thermoanaerobacterium sp. strain J-TH05 16S ribosomal RNA gene, partial sequence	100.00	1360
	Thermoanaerobacterium sp. CT387 16S ribosomal RNA gene, partial sequence	100.00	1242
	Thermoanaerobacterium sp. CT120 16S ribosomal RNA gene, partial sequence	100.00	1212
	Thermoanaerobacterium aotearoense gene for 16S ribosomal RNA, partial sequence, strain: JCA-5602	100.00	1421

Table S5 Shannon diversity index of microbial communities in reactors R1, R2 and R3 at different substrate concentrations. Reactors R1, R2 and R3 were fed with 18.2 g COD·L^{-1} of glucose, 7.8 g COD·L^{-1} of glucose, and 21.4 g COD·L^{-1} of xylose, respectively. For reactor R1, samples 610, 240, and ≈0 correspond to days 42, 91, and 142, respectively. For reactor R2, these correspond to days 42, 76, and 106, and for reactor R3 to days 51, 139, and 243, respectively

Sample	Shannon
**R1_610**	2.09396311
**R1_240**	1.44922854
**R1_0**	1.043787627
**R2_240**	2.039142354
**R2_100**	1.626126963
**R2_0**	1.013786454
**R3_670**	2.217237666
**R3_270**	1.904395478
**R3_0**	1.980767799
